# Methods to estimate the between‐study variance and its uncertainty in meta‐analysis†

**DOI:** 10.1002/jrsm.1164

**Published:** 2015-09-02

**Authors:** Areti Angeliki Veroniki, Dan Jackson, Wolfgang Viechtbauer, Ralf Bender, Jack Bowden, Guido Knapp, Oliver Kuss, Julian PT Higgins, Dean Langan, Georgia Salanti

**Affiliations:** ^1^ Li Ka Shing Knowledge InstituteSt. Michael's Hospital209 Victoria Street, East BuildingTorontoOntarioM5B 1T8Canada; ^2^ MRC Biostatistics UnitInstitute of Public HealthRobinson WayCambridgeCB2 0SRUK; ^3^ Department of Psychiatry and Psychology, School for Mental Health and NeuroscienceMaastricht UniversityThe Netherlands; ^4^ Department of Medical BiometryInstitute for Quality and Efficiency in Health Care (IQWiG)Im Mediapark 850670CologneGermany; ^5^ MRC Biostatistics Unit Hub for Trials Methodology ResearchCambridgeUK; ^6^ Department of StatisticsTU Dortmund University44221DortmundGermany; ^7^ Institute for Biometrics and Epidemiology, German Diabetes CenterLeibniz Institute for Diabetes Research at Heinrich Heine University40225DüsseldorfGermany; ^8^ School of Social and Community MedicineUniversity of BristolBristolUK; ^9^ Centre for Reviews and DisseminationUniversity of YorkYorkUK; ^10^ Department of Hygiene and EpidemiologyUniversity of Ioannina School of MedicineIoanninaGreece

**Keywords:** heterogeneity, mean squared error, bias, coverage probability, confidence interval

## Abstract

Meta‐analyses are typically used to estimate the overall/mean of an outcome of interest. However, inference about between‐study variability, which is typically modelled using a between‐study variance parameter, is usually an additional aim. The DerSimonian and Laird method, currently widely used by default to estimate the between‐study variance, has been long challenged. Our aim is to identify known methods for estimation of the between‐study variance and its corresponding uncertainty, and to summarise the simulation and empirical evidence that compares them. We identified 16 estimators for the between‐study variance, seven methods to calculate confidence intervals, and several comparative studies. Simulation studies suggest that for both dichotomous and continuous data the estimator proposed by Paule and Mandel and for continuous data the restricted maximum likelihood estimator are better alternatives to estimate the between‐study variance. Based on the scenarios and results presented in the published studies, we recommend the Q‐profile method and the alternative approach based on a ‘generalised Cochran between‐study variance statistic’ to compute corresponding confidence intervals around the resulting estimates. Our recommendations are based on a qualitative evaluation of the existing literature and expert consensus. Evidence‐based recommendations require an extensive simulation study where all methods would be compared under the same scenarios. © 2015 The Authors. *Research Synthesis Methods* published by John Wiley & Sons Ltd.

## 1 Introduction

Meta‐analysis combines estimates of quantities of interest, as obtained from studies addressing the same research question. A degree of variability in study estimates is inevitably present because of within‐study sampling error. Additional variability might occur for many reasons such as differences in the way studies are conducted and how the treatment effects are measured; this additional variability is usually modelled using a between‐study variance parameter. Between‐study variance refers to variation across study findings beyond random sampling error, and its quantification is often of interest and aids in the interpretation of results of a meta‐analysis. Several methods have been suggested to quantify the amount of between‐study variance in meta‐analytic data. In the most popular family of methods, the between‐study variance is represented by the variance of the distribution of the true study effects, commonly denoted as *τ* ^2^ in the meta‐analytic literature.

Two approaches are most commonly applied for combining the study findings in a meta‐analysis: (1) the fixed‐effect (FE) model, and (2) the random‐effects (RE) model (for a detailed description of the models, see section 2) (Borenstein *et al*., 2009). These two models are associated with different assumptions and the selection between the two might importantly impact on meta‐analytic conclusions. In the FE model, the observed treatment effects are distributed around one common true treatment effect with distribution variance informed entirely by the within‐study variances. In the RE model, the observed treatment effects estimate different study‐specific true treatment effects, which are related and assumed to come from the same underlying distribution. The variability in the distribution is therefore attributed to both within‐study variance, because of sampling error, and between‐study variance. A different approach is the fixed‐effects model, in which the true treatment effect parameters are unknown and estimated by the observed effects as in the RE model, but are treated as fixed and unrelated constants (Gardiner *et al*., 2009; Laird and Mosteller, 1990). Although the names of the ‘fixed‐effect’ and ‘fixed‐effects’ models are so similar, the assumptions under which the models are constructed are completely different. The description of the fixed‐effects model is not presented in section 2 as it is beyond the scope of this review.

Several estimators for the between‐study variance component (*τ* ^2^) have been proposed that vary in popularity and complexity. The DerSimonian and Laird (1986) (DL) method is the most commonly implemented approach and is the default approach in many software routines. However, its default use has often been challenged in the sense that DL may underestimate the true between‐study variance, potentially producing overly narrow confidence intervals (CIs) for the mean effect (Cornell *et al*., 2014), especially when the between‐study variance is large (Novianti *et al*., 2014). The use of alternative estimation methods is in practice limited by their availability in software. In Table 1, we summarise the between‐study variance estimators currently available in various software packages.

**Table 1:** Software option (with packages or macros) for each τ^2^ estimation method. Το our knowledge, routines for Hartung and Makambi, two‐step DerSimonian and Laird, positive DerSimonian and Laird, two‐step Hedges and Olkin, Rukhin Bayes, positive Rukhin Bayes, and non‐parametric bootstrap methods are not available in any of the software options listed below. The relevant references for the underlying packages and macros are presented at the end of the table.

Software	License type	Estimation methods (packages/macros)									
		DerSimonian and Laird (DL)	Paule and Mandel (PM)	Hedges and Olkin (HO)	Hunter and Schmidt (HS)	Maximum likelihood (ML)	Restricted maximum likelihood (REML)	Approximate restricted maximum likelihood (AREML)	Sidik and Jonkman (SJ)	Full Bayes (FB)	Bayes modal (BM)
Comprehensive Meta‐Analysis (Borenstein *et al*., 2005) www.meta‐analysis.com/	Commercial	Yes	—	—	—	Yes	—	—	—	—	—
Excel using the MetaEasy AddIn (Kontopantelis and Reeves, 2009) http://www.jstatsoft.org/v30/i07	Freeware	Yes	—	—	—	Yes	—	—	—	—	—
HLM (Raudenbush *et al*., 2004) http://www.ssicentral.com/hlm/	Commercial	—	—	—	—	Yes	Yes	—	—	—	—
Meta‐DiSc (Zamora *et al*., 2006) ftp://ftp.hrc.es/pub/programas/metadisc/	Freeware	Yes	—	—	—	Yes	Yes	—	—	—	—
Metawin (Rosenberg *et al*., 2000) http://www.metawinsoft.com/	Commercial	Yes	—	—	—	Yes	—	—	—	—	—
MIX (Bax, 2011) www.mix‐for‐meta‐analysis.info/	Commercial	Yes	—	—	—	—	—	—	—	—	—
MLwin (Rasbash *et al*., 2014) http://www.bristol.ac.uk/cmm/software/mlwin/	Freeware	—	—	—	—	Yes	Yes	—	—	Yes	—
Open Meta Analyst (Wallace *et al*., 2012) http://www.cebm.brown.edu/open_meta	Freeware	Yes	Yes	Yes	—	Yes	Yes	—	Yes	—	—
RevMan (The Nordic Cochrane Centre, 2014) www.cochrane.org/	Freeware	Yes	—	—	—	—	—	—	—	—	—
R (R Development Core Team, 2008) http://www.r‐project.org/	Freeware	Yes (meta, metafor, netmeta, mvmeta)	Yes (meta, metafor)	Yes (meta, metafor, mvmeta)	Yes (meta, metafor)	Yes (meta, metaSEM, metafor, mvmeta)	Yes (meta, metaSEM, metafor, mvmeta)	—	Yes (meta, metafor)	Yes (R2WinBUGS,BRugs, rjugs)	Yes (blme)
SAS (SAS Institute Inc., 2003) http://www.sas.com/technologies/analytics/statistics/stat/	Commercial	Yes (marandom.sas)	—	—	—	Yes (marandom.sas, PROC IML, PROC MIXED, PROC GLIMMIX)	Yes (PROC IML, PROC MIXED, PROC GLIMMIX)	—	—	Yes (SASBUGS, RASmacro, PROC MCMC)	—
Stata (StataCorp, 2013) www.stata.com /	Commercial	Yes (metareg, metan, metaan, mvmeta)	Yes (metareg)	—	—	Yes (metareg, metaan, mvmeta)	Yes (metareg, metaan, mvmeta)	—	—	—	Yes (gllamm)
SPSS (IBM Corp., 2013) http://www.spss.co.in/	Commercial	Yes (meanes.sps, metaf.sps, metareg.sps)	—	—	—	Yes (metaf.sps, metareg.sps)	—	Yes (metaf.sps, metareg.sps)	—	—	—
BUGS (Thomas, 1994), OpenBUGS (Thomas, 2010), or WinBUGS (Lunn *et al*., 2000) www.mrc‐bsu.cam.ac.uk/bugs/	Freeware	—	—	—	—	—	—	—	—	Yes	—

**R**: meta (http://cran.r‐project.org/web/packages/meta/meta.pdf), metafor (Viechtbauer, 2013) (http://www.metafor‐project.org/doku.php), netmeta (http://cran.r‐project.org/web/packages/netmeta/netmeta.pdf), mvmeta (http://cran.r‐project.org/web/packages/mvmeta/mvmeta.pdf), metaSEM (http://courses.nus.edu.sg/course/psycwlm/Internet/metaSEM/), R2WinBUGS (http://cran.r‐project.org/web/packages/R2WinBUGS/R2WinBUGS.pdf), BRugs (http://cran.r‐project.org/web/packages/BRugs/BRugs.pdf), rjugs (http://cran.r‐project.org/web/packages/rjags/rjags.pdf), blme (http://cran.r‐project.org/web/packages/blme/blme.pdf)

**SAS**: marandom.sas (http://www.senns.demon.co.uk/SAS%20Macros/SASMacros.html), PROC IML (http://support.sas.com/documentation/cdl/en/imlug/63541/PDF/default/imlug.pdf), PROC MIXED (https://support.sas.com/documentation/cdl/en/statugmixed/61807/PDF/default/statugmixed.pdf), PROC GLIMMIX (https://support.sas.com/documentation/cdl/en/statugglmmix/61788/PDF/default/statugglmmix.pdf), SASBUGS (Zhang *et al*., 2008), RASmacro (https://github.com/rsparapa/rasmacro), PROC MCMC (http://support.sas.com/documentation/cdl/en/statugmcmc/63125/PDF/default/statugmcmc.pdf)

**Stata**: metareg (Harbord and Higgins, 2008), metan (Harris *et al*., 2008), metaan (Kontopantelis and Reeves, 2010), mvmeta (White, 2009), gllamm (Rabe‐Hesketh *et al*., 2003) (http://www.gllamm.org/programs.html)

**SPSS**: meanes.sps (http://mason.gmu.edu/~dwilsonb/ma.html), metaf.sps (http://mason.gmu.edu/~dwilsonb/ma.html), metareg.sps (http://mason.gmu.edu/~dwilsonb/ma.html)

Simulation studies (Chung *et al*., 2014; Kontopantelis *et al*., 2013; Sidik and Jonkman, 2007) have shown that estimates of the between‐study variance are particularly inaccurate when the number of studies included in a meta‐analysis is small. Inferences about the amount of between‐study variance can therefore be misleading when only a point estimate is considered. Confidence intervals for *τ* ^2^ can facilitate interpretation (Ioannidis *et al*., 2007). Again, several options exist to quantify the uncertainty in the estimated amount of the between‐study variance.

While a measure of the between‐study variance is arguably a core output of a meta‐analysis, investigators often consider it as an intermediate step when fitting a RE model. In particular, the inverse‐variance meta‐analysis method estimates a summary treatment effect as the weighted average of individual study findings, with weights depending on both within‐study (sampling) variance and the estimated between‐study variance. Consequently, the estimated amount of the between‐study variance influences the weights assigned to each study and hence the overall summary treatment effect and, importantly, its precision. Although it is typical to assume that the weights are known constants when constructing confidence intervals and hypothesis tests, they are in fact unknown random variables that need to be estimated. This issue has previously been discussed for the usual tests that, as a consequence, do not have the properties assumed for them, such as the chi‐square distribution for the Q‐statistic under the null hypothesis of homogeneity (Kulinskaya *et al*., 2011a, 2011b), and some attempts have been made to account for the uncertainty in the weights (Böhning *et al*., 2002; Malzahn *et al*., 2000). Hence, among other factors, the performance of the between‐study variance estimators depends on how well we estimate the study weights.

Special attention should also be paid to the case of rare events in dichotomous outcome data using the inverse‐variance approach, where the degree of bias in the between‐study estimation is proportional to the rarity of the study events. In such cases, it has been suggested to avoid the inverse‐variance method altogether and instead either use the FE model and the Mantel–Haenszel method for unbalanced group sizes or Peto's method for balanced group sizes (Bradburn *et al*., 2007; Sweeting *et al*., 2004), or to switch to models/methods based on exact distributional assumptions (Kuss, 2014; Stijnen *et al*., 2010).

In this paper, we provide a comprehensive overview of methods used for estimating the between‐study variance and its uncertainty. We searched in PubMed to identify research articles that describe or compare these methods in simulation or empirical studies, and we scanned the references of the selected articles for additional relevant literature (see Supporting Information). Eligible were methods that can be applied for any type of outcome data. Several published papers present and compare various between‐study variance estimators (Chung *et al*., 2014; DerSimonian and Kacker, 2007; Novianti *et al*., 2014; Pullenayegum, 2011; Sidik and Jonkman, 2007; Viechtbauer, 2005) and their CIs (Jackson, 2013; Knapp *et al*., 2006; Viechtbauer, 2007), while many published empirical and simulation studies suggest different methods (Chung *et al*., 2014; Kontopantelis *et al*., 2013; Novianti *et al*., 2014; Sidik and Jonkman, 2007; Thorlund *et al*., 2011; Viechtbauer, 2007). Therefore, there is a need to summarise the alternative estimation options and the studies' conclusions, and to indicate whether some methods are preferable to others with regard to the studies' results.

After a brief description of the usual meta‐analysis models in section 2, we describe 16 methods to estimate between‐study variance in section 3. We describe seven methods to quantify uncertainty in the between‐study variance in section 4, and then illustrate the application of the various methods in example meta‐analyses along with our recommendations in section 5. We conclude with a summary of the comparative studies that evaluate the performance of the various methods in section 6. Our recommendations are based on a qualitative evaluation of the existing literature and expert consensus. Evidence‐based recommendations require an extensive simulation study where all methods would be compared under the same scenarios.

## 2 Models for meta‐analysis

Consider the situation where the quantity of interest is the effect of an intervention compared to a control condition or the presence of a risk factor compared to its absence, measured in terms of an effect size (such as the log‐odds ratio or the standardised mean difference). The main two parametric models used to combine study results are the FE model and the RE model. The FE model assumes that all studies share the same (fixed) effect, that is, there is one ‘true effect’ size and all differences in the observed effects are because of sampling error. In the RE model, the effects in the studies are assumed to represent a random sample from a distribution of true treatment effects, most commonly a normal distribution. The width of the distribution describes the degree of the between‐study variance. The RE model encompasses within‐study (*v*_*i*_, index *i* refers to the *i* ^th^ study, with *i* = 1, …, *k*) and between‐study (*τ* ^2^) variation, in contrast to the FE model which includes within‐study variation only. Uncertainty in the location of the mean effect in a RE meta‐analysis depends on the magnitude of the between‐study variance, the number of studies, and the precision of the individual study estimates (Hardy and Thompson, 1996). Several approaches have been suggested to estimate a CI around the mean effect, the performance of which has been examined in simulation studies under various scenarios (Brockwell and Gordon, 2001; Hartung, 1999; Kontopantelis and Reeves, 2012).

In the presence of between‐study variance (*τ* ^2^ > 0), the RE model results in a wider CI compared with the FE model, reflecting greater uncertainty around the mean (Villar *et al*., 2001). In Table 2, we provide notation and define both models formally.

**Table 2:** Models to synthesise study results in a meta‐analysis.

Fixed‐effect model	Random‐effects model
	*y*_*i*_ = *θ*_*i*_ + *ε*_*i*_
*y*_*i*_ = *μ*_*FE*_ + *ε*_*i*_	*θ*_*i*_ = *μ*_*RE*_ + *δ*_*i*_
*ε*_*i*_ ~ *Ν*(0, *v*_*i*_)	*ε*_*i*_ ~ *Ν*(0, *v*_*i*_)
*Var*(*y*_*i*_) = *v*_*i*_	*δ*_*i*_ ~ *Ν*(0, *τ* ^2^)
*w*_*i*,*FE*_ = 1/*v*_*i*_	*Var*(*y*_*i*_) = *v*_*i*_ + *τ* ^2^
	*w*_*i*,*RE*_ = 1/(*v*_*i*_ + *τ* ^2^)

Given observed treatment effect *y*_*i*_ in study *i* = 1, …, *k* (e.g. the log‐odds ratio), the common treatment effect *μ*_*FE*_ under the FE model is estimated as

(1) $${\hat{\mu }}_{FE}=\frac{\sum w_{i,FE}y_{i}}{\sum w_{i,FE}}$$

where *w*_*i*,*FE*_ is the weight (the inverse of the variance) assigned to each study. Note that throughout the paper all summations go from *i* = 1 to *i* = *k*. Under the assumptions of the model, the summary treatment effect in (1) is the uniformly minimum variance unbiased estimator of *μ*_*FE*_ (Viechtbauer, 2005).

Under the RE model, we instead estimate *μ*_*RE*_, the mean of the distribution of true treatment effects. Let *δ*_*i*_ denote the difference between this mean and the underlying study‐specific true effect *θ*_*i*_ in a particular study. The estimated summary treatment effect ${\hat{\mu }}_{RE}$ is computed as in Equation (1) using weights appropriate to the RE model, *w*_*i*,*RE*_, as provided in Table 2. In the following sections, we will use the notation ${\hat{\mu }}_{RE}\left({\hat{\tau }}^{2}\right)$ to emphasise that the overall treatment effect depends on the estimated amount of between‐study variance. Model parameters are usually estimated under the assumption that the study variances *v*_*i*_ are known when in fact they are estimated from the observed study data. We make this assumption throughout the paper, so the distributions of statistics presented are good approximations only when the study sizes are large.

## 3 Methods to estimate the between‐study variance

We consider 16 between‐study variance estimators, and these are summarised in Table 3, along with abbreviations which we will use in the text of the sections that follow. The methods may be divided into two main groups: closed‐form (or non‐iterative) methods and iterative methods. Closed‐form methods provide a parameter estimator in a predetermined number of steps, whereas iterative methods converge to a solution when a specific criterion is met (however, some iterative methods do not always produce a result because of failure to converge). Methods may also be distinguished by whether they yield only positive values or whether they yield non‐negative estimates (i.e. ${\hat{\tau }}^{2}$ can be zero).

**Table 3:** Overview of the estimators for the between‐study variance.

Estimator	Abbreviation	Iterative/Non‐iterative	Positive/Non‐negative
*Method of moments estimators*			
DerSimonian and Laird	DL	Non‐iterative	Non‐negative
Positive DerSimonian and Laird	DLp	Non‐iterative	Positive
Two‐step DerSimonian and Laird	DL2	Non‐iterative	Non‐negative
Hedges and Olkin	HO	Non‐iterative	Non‐negative
Two‐step Hedges and Olkin	HO2	Non‐iterative	Non‐negative
Paule and Mandel	PM	Iterative	Non‐negative
Hartung and Makambi	HM	Non‐iterative	Positive
Hunter and Schmidt	HS	Non‐iterative	Non‐negative
*Maximum likelihood estimators*			
Maximum likelihood	ML	Iterative	Non‐negative
Restricted maximum likelihood	REML	Iterative	Non‐negative
Approximate restricted maximum likelihood	AREML	Iterative	Non‐negative
*Model error variance estimator*			
Sidik and Jonkman	SJ	Non‐iterative	Positive
*Bayes estimators*			
Rukhin Bayes	RB	Iterative	Non‐negative
Positive Rukhin Bayes	RBp	Iterative	Positive
Full Bayes	FB	Iterative	Non‐negative
Bayes Modal	BM	Iterative	Positive
*Bootstrap estimator*			
Non‐parametric bootstrap DerSimonian and Laird	DLb	Iterative	Non‐negative

We describe known properties of the methods in terms of bias, mean squared error (MSE), and efficiency. Bias is the difference between the expected value of the estimator and its true value, and is given by

$$\text{Bias}\left({\hat{\tau }}^{2}\right)=E\left({\hat{\tau }}^{2}\right)-\tau^{2}=E\left({\hat{\tau }}^{2}-\tau^{2}\right)\text{.}$$

Negatively or positively biased estimators lead to an under‐ or over‐estimation of the true between‐study variance. Therefore, it is desirable that an estimator for the amount of the between‐study variance satisfies $E\left({\hat{\tau }}^{2}\right)=\tau^{2}$, that is, it is unbiased.

A good estimator should not only be unbiased, but also remain unaffected as much as possible by sampling fluctuation (efficiency), that is, the extent to which the estimator takes on different values with different samples. MSE is the squared distance between the estimator and its true value:

$$MSE\left({\hat{\tau }}^{2}\right)=E\left[{\left({\hat{\tau }}^{2}-\tau^{2}\right)}^{2}\right]=Var\left({\hat{\tau }}^{2}\right)+{\left(\text{Bias}\left({\hat{\tau }}^{2}\right)\right)}^{2}\text{.}$$

If $MSE\left({\hat{\tau }}_{1}^{2}\right)<MSE\left({\hat{\tau }}_{2}^{2}\right)$, then ${\hat{\tau }}_{1}^{2}$ is said to be more efficient than ${\hat{\tau }}_{2}^{2}$. Finally, CIs produced by different estimation methods for either *τ* ^2^ or *μ* are often compared in terms of coverage probability (i.e. the proportion of times the interval includes the true value of the parameter being estimated) and width. Methods that provide narrower CIs with coverage probability close to the nominal level are preferable.

### 3.1 *DerSimonian and Laird (DL) method*

The DL estimator is possibly the most frequently used approach as it is a non‐iterative method that is simple to implement (DerSimonian and Laird, 1986). In fact, many software routines have DL as the default method to estimate the between‐study variance. The estimator is derived by equating the expected value of Cochran's *Q*‐statistic with its observed value, yielding

$$E\left(Q\right)=\tau^{2}\left(\sum w_{i,FE}-\frac{\sum w_{i,FE}^{2}}{\sum w_{i,FE}}\right)+\left(k-1\right)\text{,}$$

where *Q* is calculated based on an estimate from a FE analysis, ${\hat{\mu }}_{FE}$ with:

$$Q=\sum w_{i,FE}{\left(y_{i}-{\hat{\mu }}_{FE}\right)}^{2}=\sum \frac{{\left(y_{i}-{\hat{\mu }}_{FE}\right)}^{2}}{v_{i}}\text{.}$$

The DL estimator can therefore be obtained as

$${\hat{\tau }}_{DL}^{2}=\max\left\{0,\frac{Q-\left(k-1\right)}{\sum w_{i,FE}\;-\;\frac{\sum w_{i,FE}^{2}}{\sum w_{i,FE}}}\right\}\text{.}$$

The Cochran's *Q*‐statistic belongs to the ‘generalised Cochran between‐study variance statistics’ (DerSimonian and Kacker, 2007):

$$Q_{a}=\sum a_{i}{\left(y_{i}-{\hat{\mu }}_{a}\right)}^{2}\text{,}$$

with *a*_*i*_ representing weights assigned to each study that are equal to any positive value, and ${\hat{\mu }}_{a}=\frac{\sum a_{i}y_{i}}{\sum a_{i}}$. Similarly to DL, equating *Q*_*a*_ to its expected value

$$E\left(Q_{a}\right)=\tau^{2}\left(\sum a_{i}-\frac{\sum a_{i}^{2}}{\sum a_{i}}\right)+\left(\sum a_{i}v_{i}-\frac{\sum a_{i}^{2}v_{i}}{\sum a_{i}}\right)\text{,}$$

and solving for *τ* ^2^ we can obtain the generalised method of moments (GMM) estimator:

$${\hat{\tau }}_{GMM}^{2}=\max\left\{0,\frac{Q_{a}-\left(\sum a_{i}v_{i}-\frac{\sum a_{i}^{2}v_{i}}{\sum a_{i}}\right)}{\sum a_{i}-\frac{\sum a_{i}^{2}}{\sum a_{i}}}\right\}\text{.}$$

Therefore, the DL estimator is a special case of the general class of method of moments estimators with weights *a*_*i*_ = *w*_*i*,*FE*_ = 1/*v*_*i*_.

Under the assumptions of the RE model assuming known within‐study variances *v*_*i*_ and before the truncation of negative values, the generalised method of moments estimator is unbiased. However, the need to truncate negative values to zero introduces positive bias into the estimator (Rukhin, 2013; Viechtbauer, 2005) and consequently the DL estimator is positively biased, over‐estimating the true amount of between‐study variance on average. When *k* decreases and/or the *v*_*i*_ increase, the estimator becomes more variable and truncation is often needed, leading to positive bias (Viechtbauer, 2005).

However, before truncation, the DL estimator is unbiased if the sampling variances are known rather than estimated. Any bias in the estimator is therefore not inherent to the estimator per se, but depends on how well sampling variances are estimated. Potential bias in the DL estimator might also stem from other factors apart from estimation of the *v*_*i*_ values, such as bias in the treatment effect estimates and/or correlation between the treatment effect estimates and their corresponding sampling variances. It should be noted that DerSimonian and Laird (1986) suggested the truncated estimator as shown above, and from now on (unless stated otherwise) we will refer to the truncated version of their estimator as DL.

Simulation studies have suggested that the DL between‐study estimate is acceptable when true levels of the between‐study variance are small or close to zero and *k* is large, whereas when *τ* ^2^ is large, the DL estimator can produce estimates with significant negative bias (Bowden *et al*., 2011; Novianti *et al*., 2014; Sidik and Jonkman, 2007, 2005a, 2005b). The negative bias that has been reported with respect to the DL estimator seems to be something especially related to using effect size measures based on 2×2 table data (e.g. odds ratios, risk ratios), where problems arise when using very large *τ* ^2^ values in simulation studies. In particular, very large *τ* ^2^ can lead to extreme values of the effect size measure, at which point many tables will include zero cells and the accuracy and applicability of the inverse‐variance method becomes questionable.

The DL estimator is associated with lower MSE than the HO, SJ, and PM estimators (which we describe below) when the true between‐study variance is not too large (Sidik and Jonkman, 2007). Jackson *et al*. (2010) evaluated the efficiency of the DL estimator asymptotically, that is, they assessed whether the variance of this estimator attains the Cramér–Rao bound for *k* → ∞.They showed that DL is inefficient when the studies included in the meta‐analysis are of different sizes and particularly when *τ* ^2^ is large. However, they suggested that the DL estimator can be efficient for inference on *μ* when the number of studies included in the meta‐analysis is large.

### 3.2 *Positive DerSimonian and Laird (DLp) method*

Kontopantelis *et al*. (2013) carried out an empirical and a simulation study for dichotomous outcome data and concluded that ${\hat{\tau }}_{DL}^{2}$ is often estimated to be zero when its true value is positive, especially for small *k*. They claimed that positive estimation methods are preferable to non‐negative methods and proposed an alternative approach to the DL method (which we denote DLp) that ensures a positive value:

$${\hat{\tau }}_{DLp}^{2}=\left\{\begin{array}{c} {\hat{\tau }}_{DL}^{2}\;,\;{\hat{\tau }}_{DL}^{2}>0\\ \;c\;,\;{\hat{\tau }}_{DL}^{2}\leq 0 \end{array}\right.$$

with *c* denoting an arbitrary positive constant. In a simulation study reflecting the meta‐analysis of log‐odds ratios, the authors selected *c* = 0.01 and showed that DLp has lower bias than other positive estimators (SJ, HO, RBp), irrespective of the distribution of the study‐specific true effects *θ*_*i*_.

### 3.3 *Two‐step estimator with DerSimonian and Laird (DL2) method*

DerSimonian and Kacker (2007) proposed a non‐iterative, two‐step estimator (DL2). It is based on the generalised method of moments estimator and the generalised Cochran between‐study variance statistic, with $a_{i}=w_{i,RE}=1/\left(v_{i}+{\hat{\tau }}_{DL}^{2}\right)$, and ${\hat{\mu }}_{RE}\left({\hat{\tau }}_{DL}^{2}\right)$ computed as in (1) using these RE weights. The estimator can be obtained by

(2) $${\hat{\tau }}_{DL2}^{2}=\max\left\{0,\frac{Q_{w_{RE}}-\left(\sum w_{i,RE}v_{i}-\frac{\sum w_{i,RE}^{2}v_{i}}{\sum w_{i,RE}}\right)}{\sum w_{i,RE}-\frac{\sum w_{i,RE}^{2}}{\sum w_{i,RE}}}\right\}\text{.}$$

When all of the sampling variances are equal to each other, the estimator reduces to the HO method. DerSimonian and Kacker (2007) compared the DL, HO, DL2, HO2, and PM methods (which we describe below) and showed that the DL2 estimator approximates the PM estimator, which may have some desirable statistical properties. However, Bhaumik *et al*. (2012) found that for rare events the DL2 estimator is downwardly biased.

### 3.4 *Non‐parametric bootstrap DerSimonian and Laird method (DLb)*

Kontopantelis *et al*. (2013) suggested a non‐parametric bootstrap version of the DL method (DLb) by randomly sampling *B* sets of studies with replacement. In each set, they estimate *τ* ^2^ using the DL method and then estimate ${\hat{\tau }}_{DLb\;}^{2}$ as the mean of these *B* estimates. The authors carried out a simulation study and suggested that DLb was associated with lower bias than SJ or RBp (which we describe below) for *k* ≥ 5. The same study suggested that DLb performed better compared to DL in terms of identifying the presence of between‐study variance, especially when the number of studies was small. However, non‐parametric bootstrap methods perform well only when a large number of studies is included in the meta‐analysis and the observed benefit may be artificial. The study also showed that DLb revealed greater bias compared with DL, which was more profound in small meta‐analyses. Although described for the DL method, this method can in fact be employed for every between‐study variance estimator.

### 3.5 *Hedges and Olkin (HO) method*

The HO (Hedges and Olkin, 1985) estimator (also known as Cochran estimator or variance component type estimator) was first introduced in a RE analysis of variance context by Cochran (1954). Hedges (1983) discussed the estimation method for the between‐study variance component in the meta‐analytic context. The estimator is obtained by setting the sample variance

$$S_{y}^{2}=\frac{1}{k-1}\sum {\left(y_{i}-\bar{y}\right)}^{2}$$

equal to its expected value and solving for *τ* ^2^, which yields

$${\hat{\tau }}_{HO}^{2}=\max\left\{0,\frac{1}{k-1\;}\sum {\left(y_{i}-\bar{y}\right)}^{2}-\frac{1}{k\;}\sum v_{i}\right\}\text{,}$$

where $\bar{y}$ is the unweighted average of *y*_*i*_. DerSimonian and Laird (1986) noted that the difference between the DL and HO method of moments estimators is that HO is based on the unweighted variance of the treatment effect estimates, whereas DL is based on their weighted variance. The method is a special case of the generalised method of moments estimator with *a*_*i*_ = 1/*k* as DerSimonian and Kacker (2007) suggested, or with *a*_*i*_ equal to any other positive constant independent of *i*. Although the HO estimator is simple to compute and does not require an iterative numerical solution, it is not widely used. However, it is worth noting that the HO estimator is exactly unbiased (before being truncated) when the sampling variances can be estimated unbiasedly (Viechtbauer, 2005). Unbiased estimates of the sampling variances can in fact be obtained for some outcome measures used in meta‐analyses (e.g. risk differences, raw mean differences, and standardised mean differences).

DerSimonian and Laird (1986) compared the HO method with the DL, ML, and REML methods. They concluded that, on average, the DL and REML methods yield slightly larger values than ML, but all three gave lower values than the HO estimator. This was corroborated by an empirical study (Thorlund *et al*., 2011) showing that the HO estimator produces on average larger estimates than the DL method. The HO method performs well in the presence of substantial between‐study variance, especially when the number of studies is large (i.e. *k* ≥ 30), but produces large MSE (Chung *et al*., 2014; Panityakul *et al*., 2013; Sidik and Jonkman, 2007; Viechtbauer, 2005). Sidik and Jonkman (2007) showed that for large *τ* ^2^ and moderate to large *k*, the HO estimator has the largest MSE in comparison with the DL, ML, REML, PM, and SJ estimators, but for large *k*, it has smaller bias than the DL, ML, and REML estimators. Friedman (2000) derived the variances of the DL and HO estimators and found that the DL estimator is more efficient than the HO estimator when *τ* ^2^ is zero, while the opposite occurs when the amount of between‐study variance is large. However, Sidik and Jonkman (2007) in their simulations concluded the opposite, namely that the DL estimator is more efficient than the HO estimator when *τ* ^2^ is large. They attribute the different conclusions to the fact that Friedman derives variances for the estimators that do not take into account the truncation of negative values.

### 3.6 *Two‐step estimator for the Hedges and Olkin (HO2) method*

The method of DerSimonian and Kacker (2007) is a two‐step estimator and belongs to the family of the generalised method of moments estimators. In the first step, we start with the HO estimator and use the weights $a_{i}=w_{i,RE}=1/\left(v_{i}+{\hat{\tau }}_{HO}^{2}\right)$ and the overall treatment effect ${\hat{\mu }}_{RE}\left({\hat{\tau }}_{HO}^{2}\right)$ as in (1). In the second step, we obtain the HO2 estimator as in (2). When all sampling variances are equal, the estimator reduces to the HO method. DerSimonian and Kacker (2007) showed that the two‐step estimators HO2 and DL2 approximate the PM estimator better than the one‐step HO and DL estimators, and suggest the use of either DL2 or HO2 when a non‐iterative method is desired.

### 3.7 *Paule and Mandel (PM) method*

Paule and Mandel (1982) proposed to profile a special form of *Q*_*a*_, with *a*_*i*_ = *w*_*i*,*RE*_ = 1/(*v*_*i*_ + *τ* ^2^), the generalised *Q*‐statistic:

$$Q_{gen}=\sum w_{i,RE}{\left(y_{i}-{\hat{\mu }}_{RE}\left(\tau^{2}\right)\right)}^{2}\sim \chi_{k-1}^{2}\text{,}$$

until *Q*_*gen*_ equals its expected value (i.e. *E*(*Q*_*gen*_) = *k* − 1). *Q*_*gen*_ is a pivotal quantity to test the null hypothesis that the true between‐study variance is equal to a certain amount $\tau_{0}^{2}\left(\geq 0\right)$, and depends on the unknown *τ* ^2^. PM is an iterative estimator that belongs to the family of the GMM estimators, where the RE weights and overall effect are simultaneously calculated using the true value of *τ* ^2^ that is part of the pivotal quantity. Similarly to the distribution of Cochran's Q‐statistic, the chi‐square distribution of *Q*_*gen*_ depends on how well the study‐specific weights, variances, and treatment effects are estimated.

The method is actually equivalent to the empirical Bayes estimator that was discussed by Morris (1983) and introduced into the meta‐analytic context by Berkey *et al*. (1995). Provided that the sampling variances and *τ* ^2^ are fixed and known and that the first two moments exist, the expectation of *Q*_*gen*_ is equal to *k* − 1 even when the underlying distributions are not normal (Rukhin, 2013). Because *Q*_*gen*_ is a monotonically decreasing function of *τ* ^2^, ${\hat{\tau }}_{MP}^{2}$ is set equal to zero when *Q*_*gen*_ < *k* − 1 for *τ* ^2^ = 0 (DerSimonian and Kacker, 2007).

Rukhin *et al*. (2000) showed that when assumptions underlying the method do not hold, the method is still more robust compared to the DL estimator, which depends on large sample sizes. Panityakul *et al*. (2013) showed that the PM estimator is approximately unbiased for large sample sizes and also provided R code for computing the PM estimator. It has been shown that the PM method has upward bias for small *k* and *τ* ^2^, whereas for large *k* and *τ* ^2^ it is downwardly biased (Sidik and Jonkman, 2007); but generally the method is less biased than its alternatives. One simulation study suggested that PM outperforms the DL and REML estimators in terms of bias (Panityakul *et al*., 2013). Novianti *et al*. (2014) compared the DL, DL2, PM, HO, REML, and SJ estimators, and showed that the PM method performed best in terms of bias for both dichotomous and continuous outcome data. Sidik and Jonkman (2007) highlighted the methodological similarity between the SJ and PM estimators, stating that differences are because of the fact that SJ is simplified to two steps and avoids zero between‐study variance estimates, and they showed that the two estimators have similar MSE. In fact, if the SJ estimator would be iterated, then it would yield the same exact value as the PM method. Although the PM estimator seems to perform well in terms of bias, Knapp and Hartung (2003) found that it is less efficient than the DL and REML estimators.

Bowden *et al*. (2011) carried out an empirical study comparing the DL and PM estimators and showed that as the between‐study variance increases, ${\hat{\tau }}_{PM}^{2}$ becomes greater than ${\hat{\tau }}_{DL}^{2}$. The authors recommend the use of ${\hat{\tau }}_{PM}^{2}$ and provide R code to obtain the estimator, based on the general algorithm of DerSimonian and Kacker (2007), who also recommended the PM estimator for its good properties.

For the meta‐analysis of log‐odds ratios, Bhaumik *et al*. (2012) proposed an improved PM estimator for rare adverse events by borrowing strength from all studies when estimating each sampling variance. They suggest instead of the conventional *v*_*i*_ to use

$$v_{i}^{*}=\frac{1}{n_{\text{it}}+1}\left[e^{-CGR_{\iota }-{\bar{y}}_{cor}+\frac{\tau^{2}}{2}}+2+e^{CGR_{\iota }+{\bar{y}}_{cor}+\frac{\tau^{2}}{2}}\right]+\frac{1}{n_{ic}+1}\left[e^{-CGR_{\iota }}+2+e^{CGR_{\iota }}\right]\;\text{,}$$

where *n*_*it*_ and *n*_*ic*_ are the number of subjects assigned to the treatment and control group, respectively, in study *i*, CGR is the control group risk, ${\bar{y}}_{cor}$ is the simple average of the *y*_*i*_ values, and *cor* is referred to as the continuity correction. In particular, the authors added a positive constant *cor* to the observed frequencies, estimated the relative treatment effect, and then determined the optimal value of *cor* so as to retain an unbiased estimate. To obtain the improved PM estimator, the authors suggested applying the same process as in PM using weights $w_{i,RE}^{*}=1/\left(\tau^{2}+v_{i}^{*}\right)$. They concluded that this improved method reduces bias compared with the DL, DL2, and PM estimators. This approach could in principle be implemented for every between‐study variance estimator.

### 3.8 *Hartung and Makambi (HM) method*

The HM method is a modification of the DL estimation method which does not require truncation to zero (Hartung and Makambi, 2003, 2002). Taking into account the quadratic form of the random variables *y*_*i*_,

$$\frac{\sum w_{i,FE}{\left(y_{i}-{\hat{\mu }}_{FE}\right)}^{2}}{\sum w_{i,FE}-\frac{\sum w_{i,FE}^{2}}{\sum w_{i,FE}}}=\frac{Q}{\sum w_{i,FE}-\frac{\sum w_{i,FE}^{2}}{\sum w_{i,FE}}}\text{,}$$

the HM method involves multiplying the quadratic form above by the factor *Q*/(2(*k* − 1) + *Q*) to ensure positivity. The estimator is therefore given by:

$${\hat{\tau }}_{HM}^{2}=\frac{Q^{2}}{\left(2\left(k-1\right)+Q\right)\left(\sum w_{i,FE}-\frac{\sum w_{i,FE}^{2}}{\sum w_{i,FE}}\right)}\text{.}$$

This is a non‐iterative method that always produces positive values. Thorlund *et al*. (2011) carried out an empirical study comparing the DL method with the HM, REML, HO, and SJ estimators, and concluded that for small to moderate true between‐study variance values, HM and SJ produce large estimates of *τ* ^2^.

### 3.9 *Hunter and Schmidt (HS) method*

The Hunter and Schmidt (2004) estimator is given by

$${\hat{\tau }}_{HS}^{2}=\max\left\{0,\frac{Q-k}{\sum w_{i,FE}}\right\}\;\text{.}$$

The HS estimation method has been shown to be negatively biased (Viechtbauer, 2005). The HS and ML estimators have similar MSEs, which in turn are lower than the MSEs of the DL, REML, and HO estimators. Nevertheless, if unbiasedness is considered to be of importance, then the HS estimator should be avoided (Viechtbauer, 2005).

### 3.10 *Maximum likelihood (ML) method*

The ML method is asymptotically efficient but requires an iterative solution (Hardy and Thompson, 1996; Thompson and Sharp, 1999). Based on the marginal distribution *y*_*i*_ ~ *Ν*(*μ*, *v*_*i*_ + *τ* ^2^) the estimate ${\hat{\tau }}_{ML}^{2}$ is obtained by maximising the log–likelihood function

$$lnL\left(\mu ,\tau^{2}\right)=-\frac{k}{2}\ln\left(2\pi \right)-\frac{1}{2}\sum \ln\left(v_{i}+\tau^{2}\right)-\frac{1}{2}\sum \frac{{\left(y_{i}-\mu \right)}^{2}}{\left(v_{i}+\tau^{2}\right)}\text{.}$$

Setting partial derivatives with respect to *μ* and *τ* ^2^ equal to zero and solving the likelihood equations for the two parameters to be estimated, the ML estimators for *μ* and *τ* ^2^ can be obtained by

(3) $${\hat{\mu }}_{RE}\left({\hat{\tau }}_{ML}^{2}\right)=\frac{\sum w_{i,RE}y_{i}}{\sum w_{i,RE}}\;\text{,}$$

(4) $${\hat{\tau }}_{ML}^{2}=\max\left\{0,\frac{\sum w_{i,RE}^{2}\left({\left(y_{i}-{\hat{\mu }}_{RE}\left({\hat{\tau }}_{ML}^{2}\right)\right)}^{2}-v_{i}\right)}{\sum w_{i,RE}^{2}}\right\}\;\text{,}$$

where $w_{i,RE}=1/\left(v_{i}+{\hat{\tau }}_{ML}^{2}\right)$. One way to perform the maximisation is to start with an initial estimate for ${\hat{\tau }}_{ML}^{2}$, which can be decided *a priori* as a plausible value of the between‐study variance, or it can be estimated with any other non‐iterative estimation method. Then the ML estimates are obtained by iterating over ${\hat{\tau }}_{ML}^{2}$ and ${\hat{\mu }}_{RE}\left({\hat{\tau }}_{ML}^{2}\right)$ until they converge and do not change from one iteration to the next. In each iteration step, a negative between‐study variance estimate is set equal to zero. The maximisation of the likelihood can also be performed using several techniques, such as the Newton–Raphson method, the method of scoring, the simplex method, or the expectation–maximisation (EM) algorithm.

A disadvantage of iterative estimators is that they depend on the choice of maximisation method, which might fail to converge to a solution, and hence the estimator does not provide an estimated *τ* ^2^ value. This is mainly because of the maximisation method selected and a potentially flat likelihood that is hard to maximise, which is more likely to happen when *k* is small. In such cases, one could apply one of the closed‐form estimators or incorporate an informative prior on the between‐study variance within a Bayesian framework (Pullenayegum, 2011; Rhodes *et al*., 2015; Turner *et al*., 2012). Likelihood‐based methods are asymptotically unbiased, with variance approaching the Cramér–Rao lower bound. Hence, the ML and REML methods are asymptotically equivalent, but not in finite samples.

Simulation studies have suggested that although the ML estimator has a small MSE, it exhibits large negative bias for large *τ* ^2^ when *k* is small to moderate and small studies are included in the meta‐analysis (Chung *et al*., 2014; Kontopantelis *et al*., 2013; Panityakul *et al*., 2013; Sidik and Jonkman, 2007; Viechtbauer, 2005). The method described above, also assumes effect estimates are normally distributed and there is currently little evidence to suggest how the ML estimator performs under non‐normal conditions. Alternative forms of the likelihood can be used to relax the normality assumption, which result in different maximum likelihood estimators, but are beyond the scope of this paper.

It has been shown that the ML method has the smallest MSE in comparison to the REML, SJ, HO, and PM methods, but exhibits the largest amount of bias among them (Chung *et al*., 2014; Sidik and Jonkman, 2007; Thompson and Sharp, 1999; Swallow and Monahan, 1984). Another simulation study (Viechtbauer, 2005) showed that the ML and HS methods have approximately the same MSE across all values of *k* and *τ* ^2^ simulated, which in turn was lower than the MSE of the DL and REML methods. However, because of its downward bias, both Panityakul *et al*. (2013) and Viechtbauer (2005) recommended avoiding the ML estimator.

### 3.11 *Restricted maximum likelihood (REML) method*

The REML method can be used to correct for the negative bias associated with the ML method. The estimate ${\hat{\tau }}_{\text{REML}}^{2}$ is produced by setting the derivative of the restricted log‐likelihood function (Raudenbush, 2009)

$$lnL\left(\tau^{2}\right)=-\frac{k}{2}\ln\left(2\pi \right)-\frac{1}{2}\sum \ln\left(v_{i}+\tau^{2}\right)-\frac{1}{2}\sum \frac{{\left(y_{i}-{\hat{\mu }}_{RE}\left({\hat{\tau }}_{ML}^{2}\right)\right)}^{2}}{\left(v_{i}+\tau^{2}\right)}-\frac{1}{2}ln\left(\sum \frac{1}{\left(v_{i}+\tau^{2}\right)}\right)\text{,}$$

with respect to *τ* ^2^ equal to zero and solving the resulting equation for *τ* ^2^. This yields

$${\hat{\tau }}_{\text{REML}}^{2}=\max\left\{0,\frac{\sum w_{i,RE}^{2}\left({\left(y_{i}-{\hat{\mu }}_{RE}\left({\hat{\tau }}_{ML}^{2}\right)\right)}^{2}-v_{i}\right)}{\sum w_{i,RE}^{2}}\;+\frac{1}{\sum w_{i,RE}}\right\}\text{,}$$

where $w_{i,RE}=1/\left(v_{i}+{\hat{\tau }}_{\text{REML}}^{2}\right)$ (DerSimonian and Laird, 1986; Sidik and Jonkman, 2007). Again, ${\hat{\tau }}_{\text{REML}}^{2}$ is calculated by a process of iteration with an initial estimate of ${\hat{\tau }}_{\text{REML}}^{2}\geq 0$. Each iteration step requires non‐negativity.

Simulation studies suggested that the REML method underestimates *τ* ^2^ especially when the data are sparse (Goldstein and Rasbash, 1996; Novianti *et al*., 2014; Sidik and Jonkman, 2007, 2005b). For dichotomous outcome data, it has been shown that the REML estimator is less downwardly biased than the DL estimator but has greater MSE (Chung *et al*., 2014; Sidik and Jonkman, 2007). Viechtbauer (2005) used continuous simulated data to compare the DL, ML, REML, HS, and HO methods and calculated bias and MSE with the non‐truncated estimates of *τ* ^2^. He showed that the REML estimator has smaller MSE than the HO estimator, larger MSE than the ML and HS estimators, and comparable MSE to the DL estimator. The same study showed that REML is the preferable approach when large studies are included in the meta‐analysis. For continuous outcomes, Novianti *et al*. (2014) also suggest that REML may be a valid alternative than the DL method. Knapp and Hartung (2003) found that the REML estimator has lower variance than the DL and PM estimators. Jackson *et al*. (2010) investigated the asymptotic efficiency of the DL, ML, and REML methods and showed that ML estimation performs better for small amounts of between‐study variance, whereas for large *τ* ^2^, the DL and REML estimators are more efficient. Chung *et al*. (2014) showed that the DL and REML methods produce similar proportions of zero estimates and lower than the ML estimator when *k* is small, but for large *k*, the DL, ML, and REML estimators are similar and lower in magnitude than the HO method. An empirical study (Thorlund *et al*., 2011) of 920 Cochrane reviews with dichotomous outcome data and meta‐analyses including at least three studies showed that the REML estimator can be smaller or larger in magnitude than the DL method. This agrees with a simulation study comparing DL with REML estimates under several non‐normal distributions for the effect measures, and suggests that REML is a computationally intensive iterative method and does not perform better than DL (Kontopantelis and Reeves, 2012).

### 3.12 *Approximate restricted maximum likelihood (AREML) method*

An approximate REML (AREML) estimate is also available and it is an iterative solution to (Morris, 1983; Sidik and Jonkman, 2007; Thompson and Sharp, 1999)

$${\hat{\tau }}_{\text{AREML}}^{2}=\max\left\{0,\frac{\sum w_{i,RE}^{2}\left(\left(\frac{k}{k-1}\right){\left(y_{i}-{\hat{\mu }}_{RE}\left({\hat{\tau }}_{\text{AREML}}^{2}\right)\right)}^{2}-v_{i}\right)}{\sum w_{i,RE}^{2}}\right\}\;\text{,}$$

where $w_{i.RE}=1/\left(v_{i}+{\hat{\tau }}_{\text{AREML}}^{2}\right)$. Although Thompson and Sharp (1999) describe the method as REML, this is an approximation of REML using a direct adjustment for the loss of degrees of freedom. The method yields almost identical estimates to REML (Sidik and Jonkman, 2007). In the scenario where the sampling variances are equal (*v*_*i*_ = *v*), AREML and REML estimates are identical.

### 3.13 *Sidik and Jonkman (SJ) method*

Sidik and Jonkman (2005b) introduced a non‐iterative estimation method based on weighted least squares. To obtain the SJ estimator (also known as the model error variance estimator) we first calculate the values ${\hat{q}}_{i}={\hat{r}}_{i}+1$ with ${\hat{r}}_{i}=v_{i}/{\hat{\tau }}_{0}^{2}$ (assuming ${\hat{\tau }}_{0}^{2}\neq 0$) where ${\hat{\tau }}_{0}^{2}=\sum {\left(y_{i}-\bar{y}\right)}^{2}/k$ is an initial estimate of the between‐study variance. Then the SJ estimator is obtained by setting the quantity $\sum {\hat{q}}_{i}^{-1}{\left(y_{i}-{\hat{\mu }}_{\hat{q},RE}\right)}^{2}$ equal to its expected value

$${\hat{\tau }}_{SJ}^{2}=\frac{1}{k-1\;}\sum {\hat{q}}_{i}^{-1}{\left(y_{i}-{\hat{\mu }}_{\hat{q},RE}\right)}^{2}\text{,}$$

where ${\hat{\mu }}_{\hat{q},RE}=\sum {\hat{q}}_{i}^{-1}y_{i}/\sum {\hat{q}}_{i}^{-1}$ is the weighted RE pooled estimate. An improvement on this estimation method has been recommended (Sidik and Jonkman, 2007), using ${\hat{r}}_{i}=v_{i}/{\hat{\tau }}_{HO}^{2}$ (if ${\hat{\tau }}_{HO}^{2}=0$, we set ${\hat{\tau }}_{HO}^{2}=0.01$). The method always yields a positive estimate of the between‐study variance.

The SJ estimator has methodological similarities with the PM estimator. As in the PM method, the weights assigned to each study when estimating ${\hat{\tau }}_{SJ}^{2}$ can be re‐expressed as ${\hat{q}}_{i}={\hat{r}}_{i}+1=\left(v_{i}+{\hat{\tau }}_{0}^{2}\right){\left({\hat{\tau }}_{0}^{2}\right)}^{-1}$, that is, RE weights multiplied by the constant term ${\hat{\tau }}_{0}^{2}$. In practice, the SJ method differs from the PM estimator in being always positive and non‐iterative.

Simulation studies suggested that the SJ estimation method has smaller MSE and substantially smaller bias than the DL estimator for large values of *k* and *τ* ^2^, whereas the opposite occurs when *k* and *τ* ^2^ are small (Sidik and Jonkman, 2007, 2005b). It was shown that the SJ method has smaller MSE compared with the HO method irrespective of the magnitude of *k* and *τ* ^2^, but that the latter performs better in terms of bias when *τ* ^2^ is small (Sidik and Jonkman, 2007). Additionally, the SJ estimator has been shown to have the largest bias among the DL, ML, REML, HO, and PM estimators for relatively small values of *τ* ^2^, with the bias decreasing as *τ* ^2^ increases (Novianti *et al*., 2014; Panityakul *et al*., 2013; Sidik and Jonkman, 2007). For large *τ* ^2^, the SJ and PM methods are the best estimators in terms of bias according to Sidik and Jonkman (2007). In agreement with these findings, an empirical study (Thorlund *et al*., 2011) showed that the SJ estimator produces larger estimates than the DL method.

### 3.14 *Rukhin Bayes (RB) method*

Under the assumption that *v*_*i*_ and *τ* ^2^ are random independent parameters and that the prior distribution of *τ* ^2^ is non‐informative with large variance and mean ${\hat{\tau }}_{\text{prior}}^{2}$, Rukhin (2013) derived the general form of Bayes estimators:

(5) $${\hat{\tau }}_{RB}^{2}=\max\left\{0,\frac{\sum {\left(y_{i}-\bar{y}\right)}^{2}}{k+1}\;+\frac{\left(\sum n_{i}-k\right)\left(2k{\hat{\tau }}_{\text{prior}}^{2}-\left(k-1\right)\sum v_{i}\right)}{\sum \left(n_{i}-k+2\right)\;k\left(k+1\right)}\right\}\text{,}$$

where *n*_*i*_ is the number of subjects in study *i* and ${\hat{\tau }}_{\text{prior}}^{2}$ is the mean of the prior distribution of *τ* ^2^. He showed that estimators of class (5) have an inherent positive bias. Rukhin (2013) recommended this estimator for small to moderate *k* and more specifically to estimate ${\hat{\tau }}_{RB}^{2}$ with ${\hat{\tau }}_{\text{prior}}^{2}=0$ (RB0) as an alternative to the DL method. Note that setting ${\hat{\tau }}_{\text{prior}}^{2}=0.5\left(k-1\right)\sum \left(v_{i}/k\right)$ results in a positive estimator (RBp). A simulation study (Kontopantelis *et al*., 2013) for *k* < 5 showed that RB0 had less bias than the DL, DLp, DLb, DL2, HO, HO2, REML, and SJ estimators.

### 3.15 *Bayes Modal (BM) method*

Chung *et al*. (2014, 2013) proposed the use of Bayes modal (BM) or maximum penalised likelihood estimators with a gamma prior *G*(2, 10^− 4^). To derive the BM estimator, they use the profile log‐likelihood:

$$lnL_{p}\left(\tau \right)=-\frac{k}{2}\ln\left(2\pi \right)-\frac{1}{2}\sum \ln\left(v_{i}+\tau^{2}\right)-\frac{1}{2}\sum \frac{{\left(y_{i}-\frac{\sum {\left(v_{i}+\tau^{2}\right)}^{-1}y_{i}}{\sum {\left(v_{i}+\tau^{2}\right)}^{-1}}\right)}^{2}}{\left(v_{i}+\tau^{2}\right)}\text{.}$$

Approximating *lnL*_*p*_(*τ*) using the ML estimator and a Taylor expansion, the BM estimator is obtained as

$${\hat{\tau }}_{BM}^{2}=\;\left\{\begin{array}{cc} Var\left({\hat{\tau }}_{ML}\right) & ,{\hat{\tau }}_{ML}=0\\ {\left(\frac{{\hat{\tau }}_{ML}}{2}+\frac{{\hat{\tau }}_{ML}}{2}\sqrt{1+\frac{4Var\left({\hat{\tau }}_{ML}\right)}{{\hat{\tau }}_{ML}^{2}}}\right)}^{2} & ,{\hat{\tau }}_{ML}>0 \end{array}\right.$$

where $Var\left({\hat{\tau }}_{ML}\right)$ represents the estimated asymptotic variance of *τ* based on the Fisher information (see also 4.2). The method always yields positive estimates and larger values than ${\hat{\tau }}_{ML}$ (2014, 2013). It has also been shown that the REML and BM estimators provide similar results for large *τ* ^2^, but for small values of between‐study variance, REML estimation underestimates *τ* ^2^ in contrast to the BM estimator. When *τ* ^2^ is zero, Chung *et al*. (2014) showed that the BM estimator overestimates the between‐study variance and has larger bias than the DL, ML, REML, and HO estimators especially when the study sizes and *k* are small. When *τ* ^2^ is positive, the BM estimator has the lowest MSE, whereas for *τ* ^2^ = 0, the BM estimator performed worse in terms of efficiency than the DL, ML, and REML estimators, but still better than the HO method.

### 3.16 *Full Bayesian (FB) method*

Estimates of the amount of between‐study variance can also be obtained with fully Bayesian (FB) approaches, using Markov chain Monte Carlo (MCMC) methods (e.g. a Gibbs sampler) in specialised software such as WinBUGS (see Table 1) (Smith *et al*., 1995). The FB approach is often preferable as it allows incorporation of uncertainty in all parameters (including *τ* ^2^), for which credible intervals can be derived from the posterior distribution not relying on asymptotic standard errors. However, several investigators claim that in practice the differences between frequentist and Bayesian approaches appear to be small (Morris, 1983; Thompson and Sharp, 1999). A simple hierarchical Bayesian model for meta‐analysis is

(6) $$\begin{array}{l} y_{i}|\theta_{i}\sim Ν\left(\theta_{i},v_{i}\right),\\ \theta_{i}|\mu \sim Ν\left(\mu ,\tau^{2}\right),\\ \mu \sim \pi_{1}\left(.\right),\\ \tau \sim \pi_{2}\left(.\right)\text{,} \end{array}$$

where *π*_1_(.) and *π*_2_(.) are prior distributions. The FB method uses non‐informative priors to approximate a likelihood‐based analysis. With many studies (large *k*) the choice of the prior distribution does not have a major influence on the results because the data dominate the analysis. However, the choice of prior distribution is important when the number of studies is small because it may impact on the estimated between‐study variance and consequently estimation of the mean treatment effect (Lambert *et al*., 2005; Senn, 2007).

A simulation study compared 13 different prior distributions for the between‐study variance and suggested that the results might vary substantially when the number of studies is small (Lambert *et al*., 2005). The study showed that, in terms of bias, none of the distributions considered performs best for all scenarios. More specifically, inverse‐gamma, uniform, and Wishart distributions for the between‐study variance perform poorly when *k* is small and produce estimates with substantial bias. The same study suggested that a uniform prior on *τ* performs better than other priors (e.g. uniform on log variance, inverse‐gamma on variance, DuMouchel prior) in terms of bias and convergence problems. An inverse‐gamma prior with small hyper‐parameters is often considered to be an approximately non‐informative prior, but it was shown that inferences can be sensitive to the choice of hyper‐parameters (Chung *et al*., 2013; Gelman, 2006). Chung *et al*. (2014) compared the BM estimator with a FB approach using the inverse‐gamma and uniform prior distributions for *τ*, and found that for small *τ* ^2^ the inverse‐gamma produces estimates with less bias and lower MSE than BM, but the opposite was observed for larger *τ* ^2^. The uniform prior had the largest bias and MSE among the three approaches. Informative priors were recently proposed for the between‐study variance when meta‐analysing (log) odds ratios (Pullenayegum, 2011; Turner *et al*., 2012) and standardised mean differences (Rhodes *et al*., 2015), and these might considerably improve estimation when few studies are included in the meta‐analysis.

## 4 Confidence intervals for the between‐study variance

### 4.1 *Profile likelihood confidence intervals (PL)*

Hardy and Thompson (1996) established the use of profile likelihood methods in meta‐analysis. The profile likelihood (PL) method is based on the log‐likelihood function and is an iterative process that provides CIs for the between‐study variance parameter taking into account the fact that *μ* needs to be estimated as well. The log likelihood ratio statistic under the null hypothesis *H*_0_ : *τ* ^2^ = 0 is (Hardy and Thompson, 1996):

$$U=-2ln\left[\frac{L\left({\hat{\mu }}_{RE}\left(0\right),0\right)}{L\left({\hat{\mu }}_{RE}\left({\hat{\tau }}_{ML}^{2}\right),{\hat{\tau }}_{ML}^{2}\right)}\right]\sim \chi_{1}^{2}\text{.}$$

We denote ${\hat{\mu }}_{RE}\left({\tilde{\tau }}^{2}\right)$ as the value estimated by formula (3) where ${\tilde{\tau }}^{2}$ is used to calculate the RE model's study weights. A 95% CI for *τ* ^2^ can then be obtained by the set of *τ* ^2^ values satisfying (Jackson *et al*., 2010; Viechtbauer, 2007):

$$lnL\left({\hat{\mu }}_{RE}\left({\tilde{\tau }}^{2}\right),{\tilde{\tau }}^{2}\right)>lnL\left(\;{\hat{\mu }}_{ML},{\hat{\tau }}_{ML}^{2}\right)-\frac{3.84}{2}\text{.}$$

The method requires non‐negativity at each iteration step. Viechtbauer (2007) found that profile likelihood CIs based on the restricted log‐likelihood improves the coverage probability.

When *τ* ^2^ = 0, the method produces large CIs with very high coverage probabilities, whereas as *τ* ^2^ increases, the coverage probabilities approach the nominal level (Viechtbauer, 2007). This is probably because the asymptotic distribution of the likelihood ratio statistic when *τ* ^2^ = 0 is not $\chi_{1}^{2}$, but a mixture of $\chi_{1}^{2}$ and a probability mass of a random variable centred at zero (Viechtbauer, 2007). The method is implemented in Stata using the *metaan* (Kontopantelis and Reeves, 2010) command.

### 4.2 *Wald‐type confidence intervals (Wt)*

Assuming asymptotic normality for the ML estimate, a 95% Wald‐type (Wt) CI for *τ* ^2^ can be obtained as (Biggerstaff and Tweedie, 1997):

$${\hat{\tau }}_{ML}^{2}\pm 1.96\sqrt{Var\left({\hat{\tau }}_{ML}^{2}\right)}\text{.}$$

The normal asymptotic distribution for the ML method with mean *τ* ^2^ and variance equal to the inverse of the Fisher information suggests that

$$Var\left({\hat{\tau }}_{ML}^{2}\right)=2{\left(\sum w_{i,RE}^{2}\right)}^{-1}$$

and

$${\hat{\tau }}_{\text{REML}}^{2}\pm 1.96\sqrt{Var\left({\hat{\tau }}_{\text{REML}}^{2}\right)}\text{,}$$

with

$$Var\left({\hat{\tau }}_{\text{REML}}^{2}\right)=2{\left(\sum w_{i,RE}^{2}-2\frac{\sum w_{i,RE}^{3}}{\sum w_{i,RE}}+\frac{{\left(\sum w_{i,RE}^{2}\right)}^{2}}{{\left(\sum w_{i,RE}\right)}^{2}}\right)}^{-1}\text{.}$$

Because ML and REML estimates require non‐negativity, the Wt CIs should always be non‐negative. Therefore, a negative lower bound is truncated to zero. It is worth noting that the Wt CIs require a large *k* to perform well, as between‐study variances are skewed and do not follow a normal distribution.

The method produces inaccurate CIs when the distributions of ${\hat{\tau }}_{ML}^{2}$ and ${\hat{\tau }}_{\text{REML}}^{2}$ are not adequately approximated by normal distributions (Stern and Welsh, 2000), which will be the case unless *k* is large (Goldstein, 1995). Therefore, for *τ* ^2^ > 0, the Wt CIs are not expected to yield adequate coverage probabilities, whereas for *τ* ^2^ = 0 the coverage probabilities are well above the nominal 95% level (Viechtbauer, 2007). The Wt CI is implemented in Stata using the *xtreg* (Gutierrez *et al*., 2001) and in R using *metaSEM* package (Cheung, 2014).

### 4.3 *Biggerstaff, Tweedie, and Jackson confidence intervals (BT, BJ, and Jackson)*

#### 4.3.1 *Biggerstaff and Tweedie (BT) CI*

Biggerstaff and Tweedie (1997) suggested to approximate the distribution of the Cochran's Q‐statistic, $Q=\left(S_{1}-\left(S_{2}/S_{1}\right)\right){\hat{\tau }}_{DL}^{2}+\left(k-1\right)$, where $S_{r}=\sum w_{i,FE}^{r}$ (see also section 3.1), using a gamma distribution with shape and scale parameters *r* and *λ* respectively. Using the RE model, they obtain

$$E\left(Q\right)=\left(S_{1}-\frac{S_{2}}{S_{1}}\right)\tau^{2}+\left(k-1\right)\text{,}$$

$$Var\left(Q\right)=4\left(S_{1}-\frac{S_{2}}{S_{1}}\right)\tau^{2}+2\left(S_{2}-2\frac{S_{3}}{S_{1}}+\frac{S_{2}^{2}}{S_{1}^{2}}\right)\tau^{4}+2\left(k-1\right)\text{.}$$

It follows that *E*(*Q*) = *rλ* and *Var*(*Q*) = *rλ* ^2^, *r*(*τ* ^2^) = (*E*(*Q*))^2^/*Var*(*Q*), and *λ*(*τ* ^2^) = *Var*(*Q*)/*E*(*Q*). Denoting with *f*(*x*|*r*(*τ* ^2^)) the density function of the gamma distribution with shape parameter *r*(*τ* ^2^) and scale parameter 1, the 95% Biggerstaff and Tweedie (BT) CI can be obtained as the solutions of the equations:

$$\left(\int_{\frac{Q}{\lambda \left({\tilde{\tau }}^{2}\right)}}^{\infty }f\left(\left.x\right|r\left({\tilde{\tau }}^{2}\right)\right)dx=0.025,\;\int_{0}^{\frac{Q}{\lambda \left({\tilde{\tau }}^{2}\right)}}f\left(\left.x\right|r\left({\tilde{\tau }}^{2}\right)\right)dx=0.025\right)\text{,}$$

To solve these, an iterative procedure is followed as *τ* ^2^ varies, and non‐negativity is required at each iteration step. In the case that $\int_{0}^{Q/\lambda \left({\tilde{\tau }}^{2}\right)}f\left(\left.x\right|r\left({\tilde{\tau }}^{2}\right)\right)dx<$ 0.025, the interval is set equal to the null set. Obviously, this method depends on how well the gamma distribution approximates the true distribution of *Q*. SAS code to obtain the CI was provided by the authors (Biggerstaff and Tweedie, 1997).

#### 4.3.2 *Biggerstaff and Jackson (BJ) CI*

An extension of the BT CI was later proposed by Biggerstaff and Jackson (2008) (BJ), using the cumulative distribution function of *Q*, *F*_*Q*_(*x*; *τ* ^2^). The authors noted that the non‐truncated version of the DL estimator is a linear function of *Q*, and that the HM estimator is a simple function of *Q*. Thus, they express the cumulative distribution function *F*_*Q*_(*x*; *τ* ^2^) as:

$$F_{{\hat{\tau }}_{DL}^{2}}\left(x;\tau^{2}\right)=F_{Q}\left(s{\hat{\tau }}_{DL}^{2}+k-1;\tau^{2}\right)$$

for the untruncated DL estimator and

$$F_{{\hat{\tau }}_{HM}^{2}}\left(x;\tau^{2}\right)=F_{Q}\left(s\frac{x}{2}+\sqrt{2\left(k-1\right)sx+{\left(\frac{sx}{2}\right)}^{2}}\right)-F_{Q}\left(s\frac{x}{2}-\sqrt{2\left(k-1\right)sx+{\left(\frac{sx}{2}\right)}^{2}}\right)$$

for the HM estimator of *τ* ^2^ with $s=\sum w_{i,FE}-\left(\sum w_{i,FE}^{2}/\sum w_{i,FE}\right)$. A 95% CI for the between‐study variance can be obtained as the solutions of the equations (Biggerstaff and Jackson, 2008):

$$\left(1-F_{Q}\left(x;\tau^{2}\right)=0.025,\;F_{Q}\left(x;\tau^{2}\right)=0.025\right)\text{.}$$

When 1 − *F*_*Q*_(*x*; *τ* ^2^ = 0) > 0.025 the lower bound of CI is set equal to zero.

The cumulative distribution function *F*_*Q*_(*x*; *τ* ^2^) may be computed using Farebrother's algorithm (Farebrother, 1984) for positive linear combination of chi‐squared random variables. The method is implemented in R using the *CompQuadForm* (Jackson, 2013) and *metaxa* packages (Preuß and Ziegler, 2014). Preuß and Ziegler (2014) presented a simplified version of the method that does not require the calculation of the density of Cochran's Q, but only the calculation of the cumulative distribution function, which reduces computation time.

#### 4.3.3 *Jackson CI*

A generalisation of the BJ CI was recently suggested by Jackson (2013) and uses the *Q*_*a*_ statistic (see section 3.1). One option is to use *a*_*i*_ = *w*_*i*,*FE*_ but other weighting schemes are also possible. Jackson (2013) showed that *Q*_*a*_, like *Q*, is distributed as a linear combination of *χ* ^2^ random variables so that methods similar to Biggerstaff and Jackson can be used. The cumulative distribution function of $Q_{a}\left(F_{Q_{a}}\left(x;\tau^{2}\right)\right)$ is a continuous and strictly decreasing function of *τ* ^2^ and Jackson suggested obtaining the 95% CI as:

$$\left(1-F_{Q_{a}}\left(x;\tau^{2}\right)=0.025,\;F_{Q_{a}}\left(x;\tau^{2}\right)=0.025\right)$$

In the case that $F_{Q_{a}}\left(x;\tau^{2}=0\right)<0.025$, the interval is equal to the null or empty set. In such cases, Jackson (2013) suggests presenting the interval [0, 0] and recommends interpreting the finding as ‘the data appear to be highly homogeneous’ or ‘the interval estimation fails’. If $1-F_{Q_{a}}\left(x;\tau^{2}=0\right)>0.025$, the lower bound of CI is set equal to zero.

Jackson (2013) also investigated weights of the form *a*_*i*_ = 1/(*v*_*i*_ + *x*)^*p*^ and carried out a simulation study where *x* and *p* are constants. He showed that the *Q*‐profile (QP) method provides narrower CIs than BJ method for large *τ* ^2^, and vice versa for small *τ* ^2^. For moderate *τ* ^2^, he recommends using the Jackson method with weights equal to the reciprocal of the within‐study standard errors $\left(a_{i}=1/\sqrt{v_{i}}\right)$, i.e. *x* = 0 and *p* = 1/2. The method provides an exact CI under the RE model's assumptions. The CI approaches based on the generalised Cochran between‐study variance statistic are competitors to the QP method (see section 4.4) and may provide shorter CIs. The method can be implemented in R software via the *CompQuadForm* (Duchesne and Lafaye De Micheaux, 2010) and *metafor* packages. R code is also provided by the author (Jackson, 2013).

### 4.4 *Q‐profile confidence intervals (QP)*

The QP method is based on the generalised *Q*‐statistic (*Q*_*gen*_(*τ* ^2^), see section 3.7), which follows a $\chi_{k-1}^{2}$ distribution, so that (Viechtbauer, 2007)

$$P\left(\chi_{k-1,0.025}^{2}\leq Q_{gen}\left(\tau^{2}\right)\leq \chi_{k-1,0.975}^{2}\right)=0.95\text{.}$$

Employing the inversion principle, we can derive a 95% CI for *τ* ^2^ as

$$\left(Q_{gen}\left({\tilde{\tau }}^{2}\right)=\chi_{k-1,0.975}^{2},Q_{gen}\left({\tilde{\tau }}^{2}\right)=\chi_{k-1,0.025}^{2}\right)\text{,}$$

where the two ${\tilde{\tau }}^{2}$ values are iteratively computed from $Q_{gen}\left({\tilde{\tau }}^{2}\right)$, so that the lower and upper critical values of the distribution are reached. Note that non‐negativity is required at each iteration step. If $Q_{gen}\left({\tilde{\tau }}^{2}=0\right)<\chi_{k-1,0.025}^{2}$ then the upper bound of the CI falls below zero and no CI can be provided for *τ* ^2^ resulting in the null set ([0, 0]). It is also possible that the CI does not actually contain the estimate of the between‐study variance. However, the QP method in conjunction with the PM estimator prevents such strange cases (Viechtbauer, 2013). It is also worth pointing out that under the assumptions of the RE model, the method provides an exact CI rather than an approximation.

The method has been suggested also by Hartung and Knapp (2005) for the RE model in the analysis of variance. Knapp *et al*. (2006) suggested a modified QP method to determine the lower bound of the interval using a different weighting scheme for the generalised *Q*‐statistic. For small *τ* ^2^, the modified QP is closer to the nominal level, whereas for large *τ* ^2^ the two approaches provide very similar estimates (Knapp *et al*., 2006).

Knapp *et al*. (2006) state that the QP method is preferable to the BT and PL methods with respect to attaining a predefined confidence level. Viechtbauer (2007) showed that the method guarantees nominal coverage probabilities even in small samples and that it yields the most accurate coverage probabilities among the BT, PL, Wt parametric, and the non‐parametric bootstrap methods. The method is implemented in R software in the *metafor* (Viechtbauer, 2013) package. Bowden *et al*. (2011) also provide an R function to calculate intervals using the Q‐profile method.

### 4.5 *Sidik and Jonkman confidence intervals (SJ)*

Sidik and Jonkman (2005b) proposed a method based on the SJ estimator and the 2.5^th^–97.5^th^ quantiles of the $\chi_{k-1}^{2}$ distribution:

$$\left(\frac{\left(k-1\right){\hat{\tau }}_{SJ}^{2}}{\chi_{k-1,0.975}^{2}},\frac{\left(k-1\right){\hat{\tau }}_{SJ}^{2}}{\chi_{k-1,0.025}^{2}}\right)\text{.}$$

As ${\hat{\tau }}_{SJ}^{2}$ takes non‐negative values, the interval should also be non‐negative. Simulation studies suggest that the Sidik and Jonkman (SJ) intervals have very poor coverage probability when *τ* ^2^ is small, but as *k* and *τ* ^2^ increase the coverage probability gets close to the nominal value (Sidik and Jonkman, 2005b; Viechtbauer, 2007). Consequently, Sidik and Jonkman (2005b) recommend this method only when there is strong evidence that the between‐study variance is moderate to large. However, when *τ* ^2^ = 0 the method never captures the true value and Viechtbauer (2007) raised concerns about the coverage for the estimator.

### 4.6 *Bootstrap confidence intervals*

The bootstrap approach yields parametric (Turner *et al*., 2000) and non‐parametric (Switzer *et al*., 1992) CIs. For any non‐negative estimator of *τ* ^2^, the parametric bootstrap CIs can be constructed by generating *k* values from the distribution $y_{i}\sim Ν\left({\hat{\mu }}_{RE}\left({\hat{\tau }}^{2}\right),{\hat{\tau }}^{2}+v_{i}\right)$. Then the between‐study variance is estimated based on the bootstrap sample. After repeating this process *B* times, the CI is constructed by taking the 2.5^th^ and 97.5^th^ percentiles of the distribution of ${\hat{\tau }}^{2}$ values. The non‐parametric bootstrap CIs are obtained via a similar process, where *k* studies are sampled with replacement from the observed set of *y*_*i*_ and *v*_*i*_ values. For each bootstrap sample, *τ* ^2^ can be estimated using any estimator. Repeating the process *B* times, a 95% CI is given by the 2.5^th^ and 97.5^th^ percentiles of the *B* ${\hat{\tau }}^{2}$ values. The advantage of the non‐parametric bootstrap method is that it relaxes the distributional assumptions about the observed treatment effects. However, Viechtbauer (2007) showed that bootstrap methods yield CIs with coverage probabilities that deviate substantially from the nominal level.

### 4.7 *Bayesian credible intervals*

Bayesian credible intervals for the between‐study variance can be obtained within a Bayesian framework using specialised software such as WinBUGS (Smith *et al*., 1995). As with the point estimate of the between‐study variance, the prior selection can impact a lot on the estimated credible interval when few studies are included in the meta‐analysis.

## 5 Illustrative examples

We illustrate the statistical methods discussed in this paper using data from a recent review by the MRC Clinical Trials Unit (Bowden *et al*., 2011). We select four meta‐analyses from this review, named Sarcoma (with 14 trials), Cervix2 (with 18 trials), NSCLC1 (with 17 trials), and NSCLC4 (with 11 trials). The Sarcoma meta‐analysis assessed whether adjuvant chemotherapy improves survival in patients with localised soft‐tissue sarcoma; Cervix2 assessed whether chemoradiotherapy improves survival in women with cervical cancer compared with radiotherapy; NSCLC1 evaluated the effect of cytotoxic chemotherapy on survival patients with non‐small cell lung cancer; and NSCLC4 compared supportive care plus chemotherapy with supportive care alone in patients with advanced non‐small cell lung cancer. We selected these on the basis of values of the I^2^ index, which measures the amount of between‐study variance as a percentage of the total variation in point estimates of the treatment effect (Higgins and Thompson, 2002). The four meta‐analyses represent very low (Sarcoma: I^2^ = 0%), low (Cervix2: I^2^ = 18%), moderate (NSCLC1: I^2^ = 45%), and substantial (NSCLC4: I^2^ = 75%) between‐study variance across studies. All analyses are performed with respect to the primary outcome of overall survival, and the log hazard ratio estimates were available from each trial. We combined the aggregated data in a RE model using the different estimation methods for the between‐study variance and its uncertainty.

In the Sarcoma meta‐analysis (I^2^ = 0%) only the SJ, BM, HM, RBp, and FB estimates yielded values larger than zero (Table 4). In examples with low to moderate between‐study variance, the RBp method produced the highest ${\hat{\tau }}^{2}$ value, followed by the SJ estimator. In agreement with the simulation studies, for examples with moderate and large between‐study variance (as measured by I^2^), the HS and ML methods estimated lower ${\hat{\tau }}^{2}$ values compared with the other methods. For the example with large I^2^, the SJ, HO, HO2, and RBp methods estimated large between‐study variance values with HO yielding the largest one, followed by SJ. The differences between the estimators can be explained to some extent by the different weighting schemes they use. For example, most methods include both the within‐study variances and an estimate of the between‐study variance in the weights, whereas the DL and DLp methods use only the inverse of the within‐study variances while the HO estimator uses equal weights for all trials. The PM approach yields larger estimates than the DL method for moderate to large between‐study variance; REML estimation provides values similar to the DL estimator for low to moderate between‐study variance and larger for high between‐study variance.

**Table 4:** Estimation of the between‐study variance using different methods. Four meta‐analyses are considered that represent zero (Sarcoma: I^2^ = 0%), low (Cervix2: I^2^ = 18%), moderate (NSCLC1: I^2^ = 45%), and high (NSCLC4: I^2^ = 75%) between‐study variance

	No between‐study variance	Low between‐study variance	Moderate between‐study variance	High between‐study variance
DerSimonian and Laird (DL)	0.0000	0.0148	0.0238	0.1320
Positive DerSimonian and Laird (DLp)	0.0100	0.0148	0.0238	0.1320
Two‐step DerSimonian and Laird (DL2)	0.0000	0.0130	0.0362	0.1817
Hedges and Olkin (HO)	0.0000	0.0000	0.0366	0.2243
Two‐step Hedges and Olkin (HO2)	0.0000	0.0148	0.0389	0.1932
Paule and Mandel (PM)	0.0000	0.0132	0.0393	0.1897
Hartung and Makambi (HM)	0.0170	0.0305	0.0553	0.1732
Hunter and Schmidt (HS)	0.0000	0.0100	0.0190	0.1122
Maximum likelihood (ML)	0.0000	0.0151	0.0152	0.1314
Restricted maximum likelihood (REML)	0.0000	0.0201	0.0219	0.1560
Sidik and Jonkman (SJ)	0.0691	0.0469	0.0650	0.2091
Positive Rukhin Bayes (RBp)	0.1500	0.1132	0.1199	0.1970
Full Bayes * (FB)	0.0113	0.0216	0.0256	0.1838
Bayes Modal (BM)	0.0194	0.0308	0.0293	0.1649
Non‐parametric Bootstrap DerSimonian and Laird (DLb)	0.0000	0.0120	0.0231	0.1250

* Half normal prior is used (*τ* ~ *N*(0, 10^4^), *τ* ≥ 0).

**Figure 1:**
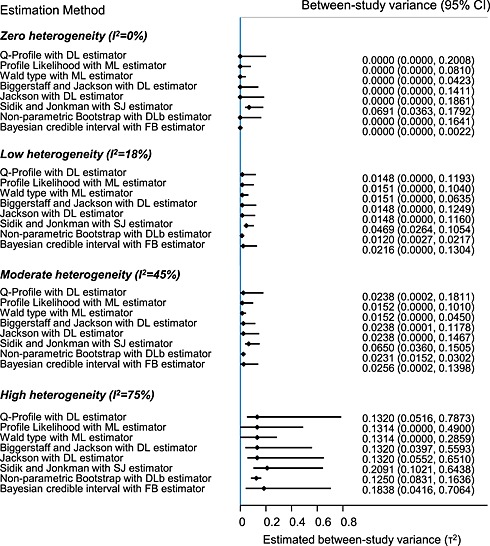
Confidence intervals for the between‐study variance for four meta‐analyses that represent zero (Sarcoma: I^2^ = 0%), low (Cervix2: I^2^ = 18%), moderate (NSCLC1: I^2^ = 45%), and high (NSCLC4: I^2^ = 75%) between‐study variance. The between‐study variance in the full Bayesian method was estimated using a half normal prior (*τ* ~ *N*(0, 10^4^), ***τ*** ≥ 0). DL: DerSimonian and Laird, DLb: Non‐parametric bootstrap DerSimonian and Laird, ML: maximum likelihood, SJ: Sidik and Jonkman, FB: Full Bayes.

In Figure 1, we present the estimated CI for *τ* ^2^ using the methods described in section 4. We display the results of the four different examples discussed above. The point estimate is calculated with the ML estimator for the PL and Wt CI methods, with the SJ estimator for the Sidik and Jonkman CI method, with the non‐parametetric DL estimator for the non‐parametric bootstrap CI method, with the FB approach for the Bayesian credible intervals, and with the DL estimator for all other CI methods. For the method suggested by Jackson (2013), we used weights equal to the reciprocal of the within‐study standard errors (i.e. with *x* = 0 and *p* = 1/2). Generally, the non‐parametric bootstrap method provides the narrowest CIs, followed by the Wt approach. In all four examples, the QP method produces comparable CIs to the BJ and Jackson methods, whereas the SJ approach always produces CIs that do not include zero.

## 6 Comparative evaluation of the methods and recommendations

We have presented 16 methods to estimate between‐study variance and seven methods to present uncertainty around the estimate. Published articles suggested that the different estimators can provide noticeably different or even conflicting results and their performance might vary regarding bias and MSE.

### 6.1 *Estimating the between‐study variance*

Selection of the most appropriate estimator might depend on (1) whether a zero value of between‐study variance is considered plausible or possible, (2) the properties of the estimators in terms of bias and efficiency, which may themselves depend on the number of studies included and the magnitude of the true between‐study variance, and (3) the ease of application (which generally favours non‐iterative methods).

The DLp, HM, SJ, RBp, and BM estimators always yield a positive estimate of the true between‐study variance. Simulation studies suggest that this results in overestimation of *τ* ^2^ when the between‐study variance is small to moderate or when the number of studies included in the meta‐analysis is small. In contrast, the non‐negative likelihood estimators and DL method tend to underestimate *τ* ^2^. Consequently, although the DL, ML, and REML estimator have smaller MSEs than the always‐positive SJ estimator, they are only recommended when the true between‐study variance in effect measures is relatively small (Sidik and Jonkman, 2007). An empirical study (Kontopantelis *et al*., 2013) showed that non‐negative methods perform well on average, but produce biased results for meta‐analyses with few studies where positive between‐study variance methods are to be preferred. Novianti *et al*. (2014) compared the estimators DL, DL2, PM, HO, REML, and SJ in a simulation study for sequential meta‐analysis when true between‐study variance is zero, and showed that all methods overestimate *τ* ^2^, with the DL, PM, and REML estimators having the best properties. Thompson and Sharp (1999) compared the DL, ML, REML, and FB estimators and concluded that the FB method may produce inflated estimates when *τ* ^2^ is close to zero and that REML estimation is the most appropriate estimation technique.

A standard assumption in meta‐analysis is that the within‐study variances are fixed and known, but in reality they need to be estimated. The non‐negative DL, HO, REML, and PM estimators are approximately unbiased (Panityakul *et al*., 2013; Viechtbauer, 2005) when the within‐study variances *v*_*i*_ are assumed to be known. The HO estimator is also approximately unbiased when the within‐study variances are not known, but can be estimated unbiasedly. Still, over‐estimates of within‐study variances can lead to negative estimates of the between‐study variance, requiring truncation. On the other hand, when the sampling variances are exactly known, truncation might also be needed for small *k*, so that estimates of *τ* ^2^ have a large variance. Truncation introduces positive bias which possibly counteracts the negative bias because of overestimation of the sampling variances. Hence, bias because of truncation depends on how well we estimate the within‐study sampling variances and the number of studies included in the meta‐analysis.

Generally, studies have found that bias increases for small number of studies and large within‐study variances. Viechtbauer (2005) recommended using REML estimation as the best approach in terms of bias and efficiency compared with the DL, ML, HS, and HO methods. The simulation study by Panityakul *et al*. (2013) suggested that the PM estimator is less biased than the DL and REML methods. Empirical evidence illustrates that the between‐study variance might vary with different effect measures (Deeks, 2002; Engels *et al*., 2000; Friedrich *et al*., 2011). It is therefore possible that the performance of the estimators might differ according to the type of outcome data. Novianti *et al*. (2014) recommended the use of the PM estimators for both dichotomous and continuous outcome data, while stated that DL2 for both outcome data and REML for continuous data are valid alternatives as well. All between‐study variance estimators described in this review can be applied with any type of outcome data and effect measure. Additional estimators have been proposed that can be applied to specific effect measures, e.g. the estimator suggested by Malzahn *et al*. (2000) and the special form of HO estimator (Hedges and Olkin, 1985), which can be applied under the standardised mean difference only.

Empirical studies have shown that most meta‐analyses using the log‐odds ratio effect measure yield *τ* ^2^ ≤ 0.4, and that the majority of meta‐analyses are informed by fewer than ten studies (Pullenayegum, 2011; Rhodes *et al*., 2015; Turner *et al*., 2012). In such cases, evidence from simulation studies shows that the HM, HO, and SJ methods overestimate *τ* ^2^ (Panityakul *et al*., 2013; Sidik and Jonkman, 2007); the BM estimator performs worse than the DL estimator (Chung *et al*., 2014); the ML method is associated with substantial negative bias (Panityakul *et al*., 2013; Sidik and Jonkman, 2007); REML estimation is less downwardly biased than the DL and ML estimators with greater MSE though (Sidik and Jonkman, 2007); and the PM estimator is less downwardly biased than the DL or REML methods (Panityakul *et al*., 2013). After joint consideration of all empirical and simulation studies, we conclude that for both dichotomous and continuous outcome data the PM estimator has good performance in most studied scenarios, and for continuous data the REML estimator appears to be preferable compared to other alternatives (Bowden *et al*., 2011; DerSimonian and Kacker, 2007; Novianti *et al*., 2014; Panityakul *et al*., 2013).

### 6.2 *Estimating confidence intervals for the between‐study variance*

Jackson (2013), Knapp *et al*. (2006), and Viechtbauer (2007) are, to the best of our knowledge, the only studies that compare methods for estimating the uncertainty around *τ* ^2^, applicable for any outcome data type. Tian (2008), suggested an additional method to the approaches presented in section 4, based on generalised inference, that can only be applied for continuous outcome data with scaled *χ* ^2^—distributed within‐study variances. The method yields adequate coverage irrespective the magnitude of the between‐study variance, and the number of studies included in a meta‐analysis. The same simulation study showed that Tian's (2008) method yields on average comparable coverage and CI length with the modified QP proposed by Knapp *et al*. (2006), but for small *τ* ^2^ values the modified QP tends to have larger CI length and coverage rate.

Viechtbauer (2007) compared the QP, PL, Wt, BT, SJ, parametric, and non‐parametric bootstrap methods. He showed that the bootstrap methods have less than adequate coverage probabilities and that the QP and BT methods yield the most accurate coverage probabilities even for small meta‐analyses (Viechtbauer, 2007). However, both the QP and BT methods can result in null sets, especially when there is low between‐study variance and the number of studies included in the meta‐analysis is small (Viechtbauer, 2007). Viechtbauer (2013) suggested employing the PM estimator of *τ* ^2^ in order to avoid estimating a CI obtained with the QP method that does not include the point estimate. The PL, Wt, and bootstrap CIs, in contrast to the QP, BT, and SJ CIs, only work well for large meta‐analyses as they are based on asymptotic results. Knapp *et al*. (2006) simulated data to compare the Wt, BT, PL, and QP CIs, and showed that the BT method was rather conservative for large *τ* ^2^, whereas the PL approach was extremely conservative for small *τ* ^2^. Jackson (2013) found that the QP method produces narrower CIs than the BJ approach for moderate to large between‐study variance, whereas for lower *τ* ^2^, CIs obtained with the BJ method should be preferable. The Jackson method with weights equal to the reciprocal of the within‐study standard errors appears to be a reasonable alternative that outperforms the QP and BJ CIs for small *τ* ^2^. However, the assumption of known and fixed within‐study variances impacts on the study weights estimation and the chi‐square approximation, and hence on the performance of these CI methods, especially for small studies.

It should be noted that the various methods for calculating confidence intervals are not all appropriate for all of the available between‐study variance estimators. In Table 5, we summarise all combinations between the approaches to estimate the uncertainty around the between‐study variance and estimators for the between‐study variance, that we suggest are likely to be considered appropriate in practice. We use three different groups to classify each pairwise combination. The classification includes combinations of approaches for estimating and calculating CIs for the between‐study variance that (1) are based on the same statistical principle and can be naturally paired, (2) could be paired in principle, but not naturally, and (3) are unlikely to be considered compatible. It is possible some pairwise combinations that we consider compatible in Table 5 yield CIs not containing the estimate of the between‐study variance, and we would not recommend presenting them together in such instances. The QP method is the default for all frequentist methods in the *metafor* package (Viechtbauer, 2013) (except when using some version of the generalised method of moments estimator for *τ* ^2^) and so this is deemed widely appropriate in Table 5; because Jackson's method is a competitor to the QP method, this alternative is deemed appropriate for the same range of possibilities as the QP method (and is used by default when using the generalised method of moments estimator). The suggestions for compatible methods for point and interval estimation in Table 5 are only tentative and we anticipate that further refinement is both possible and desirable.

**Table 5:** Summary of our proposals for appropriate combinations of approaches for estimating and calculating confidence intervals for the between‐study variance.

Between‐study variance estimators	Confidence interval for the between‐study variance methods						
	Profile Likelihood (PL)	Wald‐type (Wt)	Biggerstaff, Tweedie and Jackson (BT, BJ, Jackson)	Q‐Profile (QP)	Sidik and Jonkman (SJ)	Bootstrap	Bayesian Credible Intervals
*Method of moments estimators*							
DerSimonian and Laird (DL)	—	✓	✓	(✓)	—	✓	—
Positive DerSimonian and Laird (DLp)	—	✓	✓	(✓)	—	✓	—
Two‐step DerSimonian and Laird (DL2)	—	✓	✓	(✓)	—	✓	—
Hedges and Olkin (HO)	—	✓	✓	(✓)	—	✓	—
Two‐step Hedges and Olkin (HO2)	—	✓	✓	(✓)	—	✓	—
Paule and Mandel (PM)	—	✓	(✓)	✓	—	✓	—
Hartung and Makambi (HM)	—	✓	✓	(✓)	—	✓	—
Hunter and Schmidt (HS)	—	✓	(✓)	(✓)	—	✓	—
*Maximum Likelihood estimators*							
Maximum likelihood (ML)	✓	✓	(✓)	(✓)	—	✓	—
Restricted maximum likelihood (REML)	✓	✓	(✓)	(✓)	—	✓	—
Approximate restricted maximum likelihood (AREML)	✓	✓	(✓)	(✓)	—	✓	—
*Model error variance estimator*							
Sidik and Jonkman (SJ)	—	✓	(✓)	(✓)	✓	✓	—
*Bayes estimators*							
Rukhin Bayes (RB)	—	✓	(✓)	(✓)	—	✓	✓
Positive Rukhin Bayes (RBp)	—	✓	(✓)	(✓)	—	✓	—
Full Bayes (FB)	—	—	—	—	—	—	✓
Bayes Modal (BM)	—	✓	(✓)	(✓)	—	✓	—
*Bootstrap estimator*							
Non‐parametric bootstrap DerSimonian and Laird (DLb)	—	✓	(✓)	(✓)	—	✓	—

Pairwise combinations are categorised in three groups: a) confidence intervals naturally paired with the between‐study variance estimator, ✓; b) confidence intervals paired in principle with the between‐study variance estimator, but not naturally, (✓); c) confidence intervals considered unlikely compatible with the between‐study variance estimator, —.

### 6.3 *Recommendations*

To conclude, we offer tentative recommendations for practice based on the existing simulation and empirical studies, and informed by the consensus of the authors. The results of the available studies depend on the particular scenarios they investigated and properties of the methods they examined. Many approaches presented in this review have not been compared under the same simulation settings, and hence making any clear recommendations about these methods is difficult. Also, the selection of the most preferable methods to calculate a confidence interval for the between‐study variance is mostly based on coverage, because this was consistently reported in the identified simulation studies. Further research is required to evaluate the important properties (i.e. bias, efficiency, complexity, coverage, and precision) of all promising methods under the same, realistic, scenarios through a comprehensive simulation study.

In Appendix Table 1, we summarise scenarios examined by studies that compare between‐study variance estimators, and in Appendix Table 2, we present the results as described on average for each pairwise comparison of the estimation methods. There is limited evidence to inform which estimator performs best, in particular when the number of studies is low (<5) and when the normality assumption does not hold; and the fully Bayesian estimator has not been evaluated extensively in comparative studies. When estimation of the between‐study variance is the aim of the meta‐analysis, a sensitivity analysis using a variety of suitable methods for estimating *τ* ^2^ and its CI might be needed, particularly when studies are few in number. In Appendix Table 3, we present the simulation results with respect to the properties of all CI methods as described in the three comparative studies (Jackson, 2013; Knapp *et al*., 2006; Viechtbauer, 2007).

Overall, the popular semi‐parametric DL method appears to perform adequately. However, according to the summarised findings of the simulation studies, the PM method appears to have a more favourable profile among other estimators of the between‐study variance in meta‐analysis, including DL. It is easy to calculate, does not require distributional assumptions, (Bowden *et al*., 2011; DerSimonian and Kacker, 2007), and has been shown to be less biased and more efficient than many alternatives. For estimation of a confidence interval for the between‐study variance, a good option for large *τ* ^2^ appears to be the QP method, whereas for small *τ* ^2^ the BJ method offers a reasonable approach (Jackson, 2013). The PM method in conjunction with the QP approach for obtaining CIs fit naturally together and appear to perform satisfactorily under several realistic scenarios. For continuous outcomes, we also advocate the use of REML estimation as a preferable alternative to the DL estimator, in agreement with other recent recommendations (Novianti *et al*., 2014; Viechtbauer, 2005).

Our recommendations on the estimation of the between‐study variance are mostly based on non‐Bayesian approaches, as Bayesian estimators have not been fully investigated in simulations. However, Bayesian estimators might be considered preferable to classical estimators in situations where appropriate prior information is available and is considered suitable for use in analysis.

Our review has identified gaps and deficiencies in the existing literature, and hopefully facilitates investigators in forming their own judgements about the most appropriate method for their needs. When additional evidence becomes available, we plan to update this review and our recommendations accordingly.

## 7 Competing of interests

The authors declare that they have no competing interests.

## 8 Authors' contributors

AAV, DJ, WV, RB, JB, GK, OK, JH, DL, and GS contributed to the conception and design of the study, and helped to draft the manuscript. AAV conducted the statistical analysis. All authors read and approved the final manuscript.

## 9 Funding

AAV is funded by the CIHR Banting Postdoctoral Fellowship Program. JB is supported by an MRC Methodology Research Fellowship grant (code MR/L012286/1). DJ is employed by the UK Medical Research Council (code U105260558). DL is funded by the Centre for Reviews and Dissemination, University of York. RB is employed by the Institute for Quality and Efficiency in Health Care, Cologne, Germany. GS receives funding from the European Research Council (IMMA, grant Nr 260559).

## Supporting information



Supporting info item
