# Supplementary material for: Methods to estimate the between‐study variance and its uncertainty in meta‐analysis
Source: Res Synth Methods. 2015 Sep 2;7(1):55–79. doi: 10.1002/jrsm.1164 (PMC4950030; doi:10.1002/jrsm.1164)
Supplement: Supplementary file 1 — Supporting info item [file JRSM-7-55-s001.pdf]

Supplementary material

To identify the research articles that describe or compare the methods for the between-study variance and its uncertainty we used the following search code:

```
((heterogen*[Title/Abstract]) OR (*consisten*[Title/Abstract]) OR (between-
study variance*[Title/Abstract]) OR (between-trial variance*[Title/Abstract]))
AND (meta-analys*[Title/Abstract]) AND ((random effect*[Title/Abstract]) OR
(mixed effect*[Title/Abstract]) OR (meta-regress*[Title/Abstract])) AND
((distribution) OR (prior) OR (prediction) OR (estimat*) OR (overall treatment
effect*) OR (summary treatment effect*) OR (pooled effect*) OR (confidence
interval*) OR (bias*) OR (error*) OR (power) OR (simulation*) OR (coverage
probability*) OR (mean square* AND error*))
```

Abbreviations:

Between-study variance estimation methods	Methods for confidence intervals (CI) for between-study variance
DL: DerSimonian and Laird<br>DLp: Positive DerSimonian and Laird<br>DL2: Two-step DerSimonian and Laird<br>DLb: Non-parametric bootstrap DerSimonian and Laird<br>HO: Hedges and Olkin<br>HO2: Two-step Hedges and Olkin<br>PM: Paule and Mandel<br>HM: Hartung and Makambi<br>HS: Hunter and Schmidt<br>ML: Maximum likelihood<br>REML: Restricted maximum likelihood<br>AREML: Approximate restricted maximum likelihood<br>SJ: Sidik and Jonkman<br>RB: Rukhin Bayes<br>RBp: Positive Rukhin Bayes<br>FB: Full Bayes<br>BM: Bayes Modal	PL: Profile Likelihood CI<br>Wt: Wand-type CI<br>BT: Biggerstaff and Tweedie CI<br>BJ: Biggerstaff and Jackson CI<br>J: Jackson CI<br>B-T-J: Biggerstaff, Tweedie and Jackson CI<br>QP: Q-Profile CI<br>SJ: Sidik and Jonkman CI<br>B: Bootstrap CI<br>Bayes Cr I: Bayesian Credible Intervals

Appendix Table 1. Summary of scenarios and estimation methods, used in simulation and empirical studies that compared different estimators for the between-study variance. We denote as $logOR$ the log-odds ratio, $logRR$ the log risk-ratio, MD the mean difference, SMD the standardised mean difference, and HR the hazard ratio.

Simulation studies

Study	Scenarios	Estimation methods
	Dichotomous outcome data	
Berkey et al. [1]	$k = 10$<br>a) $logRR = -0.8387 + \delta_i$ and<br>b) $logRR = -0.8387 + 0.30x_i + \delta_i$<br>$x_i \sim N(0,1)$<br>$\delta_i \sim N(0,0.211)$	ML, REML
Bhaumik et al. [2]	$k = 20$<br>$logOR = 0,0.5,1.0, \dots ,2.5$<br>$\tau^2 = 0,0.2,0.4,0.6,0.8$	DL, DL2, PM (and improvement of PM)
Brockwell & Gordon [3]	$k = 3,4, \dots ,35$<br>$logOR = 0.5$<br>$\tau^2 = 0,0.01, \dots ,0.10$	DL, ML
Knapp & Hartung [4]	$k = 5,7,10,15$<br>$logRR = -0.5 - 0.02(x_i - \bar{x}) + \delta_i$<br>$x_i \sim N(0,1)$<br>$\delta_i \sim N(0, \tau^2)$<br>$\tau^2 = 0,0.05,0.1,0.2,0.3$	DL, REML, PM
Kontopantelis et al. [5]	$k = 5,10,20,50,100$<br>$logOR = 0.5$<br>$\tau^2 = 0.01,0.03,0.10$	DL, DL2, DLb, DLp, HO, HO2, SJ, RB0, RBp, ML, REML
Lambert et al.[6]	$k = 5, 10, 30$<br>$logOR = 0.32$<br>$\tau^2 = 0, 0.09, 0.64$	FB (inverse-gamma [(0.001,0.001), (0.1,0.1)], uniform [(1/1000,1000), (1/1000,4)], inverse-pareto [(1,0.001), (1,0.25), (0.5,0.0625)], DuMouchel on variance; uniform [(-10,10), (-10, 1.386)] on log variance; uniform [(0,100)], half-normal [(0,100), (0,1)] on standard deviation)

Novianti et al. [7]	$k = 10,15,20,30,50$<br>$logOR = 0,0.5$<br>$\tau^2 = 0,0.5, \dots ,1.5$	DL, DL2, REML, SJ (and the improvement method of SJ), HO, PM
Panityakul et al. [8]	$k = 10,30$<br>$logRR \sim \mathrm{N}(-0.37\mathrm{x_i}, \tau^2)$<br>$\mathrm{x_i} \sim \mathrm{N}(0,0.3^2)$<br>$\tau^2 = 0,(0.05),0.5$	DL, HO, PM, ML, REML, SJ
Sidik & Jonkman [9]	$k = 10,15,20,30,50$<br>$logOR = -0.50,0.00,0.50$<br>$\tau^2 = 0(0.10), 0.50(0.25), 1.75$	DL, ML, REML, SJ (and the improvement method of SJ), HO, PM
Sidik & Jonkman [10]	$k = 10,15,20,30,40,50,80,100$<br>$logOR = 0.5,1.0$<br>$\tau^2 = 0.1,0.25,0.5,0.75,1,1.25,1.5,2$	DL, SJ
	Continuous outcome data	
Chung et al.[11]	$k = 5, 10, 30$<br>$SMD = 0.5$<br>$\tau^2 = 0,0.01, 0.05, 0.1, 0.2$	DL, HO, ML, REML,BM
Chung et al.[11]	$k = 5$<br>$SMD = 0.5$<br>$\tau^2 = 0,0.01, 0.05, 0.1, 0.2$	BM, FB (uniform(0,100), and inverse-gamma(0.001, 0.001))
Chung et al.[12]	$k = 3, 5, 10, 30$<br>Meta-regression model with coefficients 0 and 1.<br>$\tau^2 = 0, 1/\sqrt{3}, 1$	ML, REML,BM
Novianti et al. [7]	$k = 10,15,20,30,50$<br>$SMD = 0,0.5$<br>$\tau^2 = 0, \dots ,0.0366$	DL, DL2, REML, SJ (and the improvement method of SJ), HO, PM
Viechtbauer [13]	$k = 5,10,20,40,80$<br>$MD = 0,1,2,4$<br>$\tau^2 = 0,0.125,0.25,0.50,1.00$	DL,ML, REML, HS, HO

Viechtbauer [13]	$k = 5,10,20,40,80$<br>$SMD = 0,0.2,0.5,0.8$<br>$\tau^2 = 0,0.01,0.025,0.05,0.10$	DL,ML, REML, HS, HO

Empirical studies		
Study	Number of studies in the meta-analysis (k)	Estimation Methods
	Dichotomous outcome data	
Biggerstaff & Tweedie [14]	9 ($P_Q < 0.01$, $logOR$ ranged from -1.47 to 1.09)	DL, ML
Biggerstaff & Tweedie [14]	32 (OR ranged from 2.55 to 0.74, $P_Q = 0.04$)	DL,ML
Brockwell & Gordon [3]	7 ($logOR$, $P_Q < 0.01$)	DL, ML
DerSimonian & Laird [15]	11 reviews with $k = 8, 9, 6, 6, 5, 26,6,6,6$.<br>Scale: $logOR$	DL, HO, ML, REML
DerSimonian & Kacker [16]	6 reviews with $k = 26, 9, 6, 3, 7, 7$<br>(pooled OR ranged from 0.14 to 0.81)	DL, DL2, HO, HO2, PM
Hardy & Thompson [17]	9 ($P_Q < 0.01$, OR ranged from 0.23 to 2.97)	DL, ML
Sidik & Jonkman [9]	29 ($logOR$ ranged from -3.00 to 2.00)	DL, ML, REML, SJ (and improved SJ), HO, PM
Sidik & Jonkman [18]	13 ($logRR$ ranged from -1.50 to 0.50)	DL, ML, REML, SJ, HO
Sidik & Jonkman [19]	9 ($logOR$ ranged from almost -1.50 to almost 1)	DL, ML, REML, SJ, HO
Thompson & Sharp [20]	a) 28 ($logOR$ ranged from -1.65 to 1.37)<br>b) 19 ($logOR$ ranged from less than 0.1 to higher than 5)	DL, ML, REML, PM, FB
Thorlund et al. [21]	920 Cochrane reviews (OR and RR)	DL, HM, HO, REML, SJ
Viechtbauer [22]	9 ($P_Q < 0.01$, $logOR$ ranged from 0.23 to 2.97)	DL, ML, REML, SJ

	Continuous outcome data	
Chung et al. [11]	10 (SMD with standard errors ranging between 0.24 and 0.57)	DL, HO, ML, REML,BM
Sanchez-Meca & Marin-Martinez [23]	10 (SMD ranged from -0.581 to 1.031)	DL, HS, HO, HM, SJ, ML, REML, the estimator proposed by Malzahn et al [24]
Sidik & Jonkman [10]	13 (SMD ranged from -2.41 to 2.93)	DL, REML, the special form of the Hedges and Olkin estimator (Hedges and Olkin, 1985, page 194) , SJ, estimator proposed by Malzahn et al [24]
Sidik & Jonkman [19]	13 (SMD ranged from around -2 to 4)	DL, ML, REML, SJ, HO
	Survival outcome data	
Bowden et al. [26]	18 me-analyses with # trials ranging between (5, 25), range of HR (0.65, 1.21), and range of I^2 (0%, 75%)	DL, PM

Appendix Table 2. Comparison of estimators for between-study variance. The diagonal summarises the main properties of the estimators. We define as k the number of studies included in the meta-analysis, and τ^2 the true between-study variance. Each off-diagonal cell compares two estimators. The lower triangle compares the estimators in terms of bias and MSE. A zero value (0) indicates that there is no difference between the two estimators, 1 indicates that the row-estimator is 'better', and -1 is 'worse'. The upper-triangle shows the references of the comparative studies. D: Dichotomous data, C: Continuous data, S: Survival data, B: Bias, MSE: Mean Squared Error

	DL	DLp	DL2	DLb	HO	HO2	PM	HM	HS	ML	REML	AREML	SJ	RB	FB	BM
**DerSimonian and Laird (DL)**		1	1;2;3;4	1	1; 3; 5; 6; 7; 8; 9; 10; 11; 12; 13; 14; 15	1; 4	2; 3; 5; 9; 15; 16; 17; 18	6; 12	6; 7	1; 5; 6; 7; 8; 10; 11; 14; 15; 17; 19; 20; 21; 22	1; 3; 5; 6; 7; 8; 10; 11; 12; 13; 14; 15; 16; 17; 22		1; 3; 5; 6; 10; 11; 12; 13; 15; 22	1	17	14
**Positive DerSimonian and Laird (DLp)**	D; B:-1		1	1	1	1				1	1		1	1		
**Two-step DerSimonian and Laird (DL2)**	D; B: 0 (small τ^2), 1 (large τ^2) | C; B: -1 (small τ^2, k), 1 (large τ^2, k)	D; B:1		1	1; 3; 4	1; 4	2; 3; 4			1	1; 3		1; 3	1		
**Non-parametric bootstrap DerSimonian and Laird (DLb)**	D; B:-1	D; B:-1	D; B:-1		1	1				1	1		1	1		
**Hedges and Olkin (HO)**	D; B:-1 (small τ^2), 1 (large τ^2); MSE: -1 (small τ^2, k), 1 (large τ^2, k) | C; B:-1 (small τ^2), 1 (large τ^2); MSE:1	D; B:-1	D; B: -1 | C; B: -1	D; B:1 (small k), -1 (large k)		1; 4	3; 4; 5; 15	6; 12	6; 7	1; 5; 6; 7; 8; 10; 11; 14; 15	1; 3; 5; 6; 7; 8; 10; 11; 12; 13; 14; 15		1; 3; 5; 6; 10; 11; 12; 15	1		14

	DL	DLp	DL2	DLb	HO	HO2	PM	HM	HS	ML	REML	AREML	SJ	RB	FB	BM
**Two-step Hedges and Olkin (HO2)**	D; B:-1	D; B:-1	D; B:-1	D; B:1 (small k), -1 (large k)	D; B:1		4			1	1		1	1		
**Paule and Mandel (PM)**	D; B: 1; MSE: -1 (small τ^2, k), 1 (large τ^2, k) | C; B: -1 (small τ^2), 1 (large τ^2) | S - Empirical study		D; B:1 | C; B:-1		D; B: 1 (small τ^2), -1 (large τ^2); MSE: 1 | C; B: 1	D - Empirical study				1; 5; 6; 7; 8; 10; 11; 15; 17	1; 3; 5; 6; 7; 8; 10; 11; 15; 16; 17		1; 3; 5; 15	1	17	
**Hartung and Makambi (HM)**	C - Empirical studies				C - Empirical studies				6	6	6; 12		6; 12			
**Hunter Schmidt (HS)**	C; B:1; MSE:1				C; B:1; MSE:1			C - Empirical study		6; 7	6; 7		6			
**Maximum Likelihood (ML)**	D; B: -1 (small τ^2, except for τ^2 values close to zero), 1 (large τ^2); MSE: 0 (small τ^2, k), 1 (large τ^2, k) | C; B:1; MSE:1	D; B:1	D; B:1	D; B:1	D; B: -1 (except for τ^2 values close to zero); MSE: -1 (small τ^2, k), 1 (large τ^2, k) | C; B:1; MSE:1	D; B:1	D; B: -1 (except for τ^2 values close to zero); MSE: -1 (small τ^2, k), 1 (large τ^2, k)	C - Empirical study	C; B:0; MSE: 1		1; 5; 6; 7; 8; 10; 11; 14; 15; 17; 22; 23; 24		1; 5; 6; 10; 11; 15; 22	1	17	14; 24

	DL	DLp	DL2	DLb	HO	HO2	PM	HM	HS	ML	REML	AREML	SJ	RB	FB	BM
**Restricted maximum likelihood (REML)**	D; B: 1; MSE: -1 (small τ^2, k), 1 (large τ^2, k) | C; B: 0 (small τ^2), 1 (large τ^2); MSE: 1	D; B:1	D; B:1 | C; B: 1 (small τ^2), -1 (large τ^2)	D; B:1	D; B: 1 (small τ^2), -1 (large τ^2); MSE: 1 (small τ^2, k), -1 (large τ^2, k) | C; B: 1 (small τ^2), -1 (large τ^2); MSE:0	D; B:1	D; B: 1 (small τ^2, k), -1 (large τ^2, k); MSE: 1 (small τ^2, k), -1 (large τ^2, k) | C; B:1 (small τ^2, k), -1 (large τ^2, k)	D | C - Empirical studies	C; B:1 MSE: -1	D; B: 1 (except for values of τ^2 close to zero); MSE: -1 (small τ^2, k), 1 (large τ^2, k) | C; B:1; MSE:-1			1; 3; 5; 6; 10; 11; 12; 13; 15; 22	1	17	14; 24
**Approximate restricted maximum likelihood (AREML)**																
**Sidik and Jonkman (SJ)**	D; B: -1 (small τ^2), 1 (large τ^2); MSE: -1 (small τ^2, k), 1 (large τ^2, k) | C; B: -1*	D; B:-1	D; B:-1| C; B:-1	D; B:1 (small k), -1 (large k)	D; B: -1 (small τ^2), 1 (large τ^2); MSE: -1 (small τ^2, k), 1 (large τ^2, k) | C; B: -1*	D; B-1	D; B: -1 (small τ^2), 0 (large τ^2); MSE: 0 | C; B: -1	D | C - Empirical studies	C - Empirical study	D; B: -1 (small τ^2), 1 (large τ^2); MSE: -1 (small τ^2, k), 1 (large τ^2, k)	D; B: -1 (small τ^2), 1 (large τ^2); MSE: -1 (small τ^2, k), 1 (large τ^2, k)| C; B: -1 (small τ^2), 1 (large τ^2)			1		
**Positive Rukhin Bayes (RBp)**	D; B:1 (small k), -1 (large k)	D; B:1 (small k), -1 (large k)	D; B:1 (small k), -1 (large k)	D; B:1 (small k), -1 (large k)	D; B:1 (small k), -1 (large k)	D; B:1 (small k), -1 (large k)				D; B:-1	D; B:-1		D; B:1			
**Full Bayes (FB)**	D - Empirical study						D - Empirical study			D - Empirical study	D - Empirical study					14

	DL	DLp	DL2	DLb	HO	HO2	PM	HM	HS	ML	REML	AREML	SJ	RB	FB	BM
**Bayes Modal (BM)**	C; B: -1 (small τ^2), 1 (large τ^2); MSE: -1 (small τ^2, k), 1 (large τ^2, k)				C; B: -1 (small τ^2, k), 1 (large τ^2, k); MSE: 1					C; B: -1 (small τ^2), 1 (large τ^2); MSE: -1 (small τ^2, k), 1 (large τ^2, k)	C; B: -1 (small τ^2), 1 (large τ^2); MSE: -1 (small τ^2, k), 1 (large τ^2, k)				C; B:-1 (small τ^2), 1 (large τ^2) ;MSE:-1 small τ^2, 1 large τ^2)	

1: Kontopantelis et al., 2013; 2: Bhaumik et al., 2012; 3: Novianti et al., 2014; 4: DerSimonian and Kacker, 2007; 5: Sidik and Jonkman, 2007; 6: Sánchez-Meca and Marín-Martínez, 2008; 7: Viechtbauer, 2005; 8: DerSimonian and Laird, 1986; 9: DerSimonian and Kacker, 2007; 10: Sidik and Jonkman, 2005a; 11: Sidik and Jonkman, 2006; 12: Thorlund et al., 2011; 13: Sidik and Jonkman, 2005b; 14: Chung et al, 2014; 15: Panityakul et al., 2013; 16: Knapp and Hartung, 2003; 17: Thompson and Sharp, 1999; 18: Bowden et al., 2011; 19: Brockwell and Gordon, 2001; 20: Biggerstaff and Tweedie, 1997; 21: Hardy and Thompson, 1996; 22: Viechtbauer, 2007; 23: Berkey et al., 1995; 24: Chung et al, 2013 – the design of the studies is described in Appendix Table 1

* For Improved SJ and large τ^2 and k, then 'B:1'

Appendix Table 3. Comparison of the confidence interval (CI) estimation methods for between-study variance (τ^2) in terms of coverage probability and CI length. A zero value (0) denotes there is no difference between the two CIs, 1 that the row-CI is better, and -1 the column-CI is better. The upper-triangle shows the references of the comparative studies. k: number of studies included in the meta-analysis

	**Profile-Likelihood (PL)**	**Wald-type (WT)**	**Q-Profile (QP)**	**Biggerstaff-Tweedie-Jackson (B-T-J)**	**Sidik-Jonkman (SJ)**	**Bootstrap (B)**	**Bayesian (Bayes) Credible Intervals (CrI)**
**PL**		Viechtbauer, 2007; Knapp et al., 2006	Viechtbauer, 2007; Knapp et al., 2006	Viechtbauer, 2007; Knapp et al., 2006	Viechtbauer, 2007	Viechtbauer, 2007	
**WT**	coverage probability: -1		Viechtbauer, 2007; Knapp et al., 2006	Viechtbauer, 2007; Knapp et al., 2006	Viechtbauer, 2007	Viechtbauer, 2007	
**QP**	coverage probability: -1	coverage probability: 1		Viechtbauer, 2007; Knapp et al., 2006; Jackson, 2013	Viechtbauer, 2007	Viechtbauer, 2007	
**B-T-J**	coverage probability:1	coverage probability: 1	coverage probability: 1 (small τ^2), -1 (large τ^2); CI length: 1 (small τ^2), -1 (large τ^2)		Viechtbauer, 2007	Viechtbauer, 2007	
**SJ**	coverage probability:-1	coverage probability:0	coverage probability: -1	coverage probability: -1		Viechtbauer, 2007	
**B**	coverage probability:-1	coverage probability:-1	coverage probability:-1	coverage probability:-1	coverage probability:1		
**Bayes CrI**							

Viechtbauer, 2007: $k = (10,20,30,50,80)$, $log - odds\ ratio = 0.5$, $\tau^2 = (0,0.1,0.2,\ldots,0.5)$

Knapp et al., 2006: $k = 5, 10, 20, 50, 100$, $mean\ difference = 0$, $\tau^2 = (1, 2.5, 5, 10, 20)$

Jackson, 2013: $k = 5$, $log - odds\ ratio = 0$, $\tau^2 = (0,0.029,0.069,0.206,1.302)$

Data used for the empirical example (see section 5)

Study	Log-Hazard Ratio	Standard Error of Log-Hazard Ratio	Study	Log-Hazard Ratio	Standard Error of Log-Hazard Ratio
	Sarcoma			***NSCLC1***	
1	-0.05184	0.194514	1	0.127128	0.083531
2	-0.36185	0.34001	2	0.052326	0.107833
3	0.226357	0.39375	3	0.322259	0.23531
4	-0.61811	0.627456	4	0.150004	0.085271
5	0.118386	0.299476	5	0.646999	0.31895
6	-0.2399	0.48737	6	-0.00622	0.262885
7	-0.01264	0.145141	7	0.580106	0.296695
8	0.046584	0.557278	8	-0.16888	0.16639
9	-0.27505	0.461757	9	-0.45556	0.215716
10	-0.87097	0.38292	10	-0.05291	0.170971
11	-0.04565	0.187383	11	-0.06485	0.584206
12	-0.63439	0.32987	12	-0.638	0.286182
13	-0.27984	0.32075	13	-0.14599	0.174395
14	0.798969	0.717958	14	0.326781	0.495682
			15	-0.33391	0.208787
			16	-0.10841	0.256326
			17	0.035793	0.19111
	Cervix2			***NSCLC4***	
1	-0.3731	0.268899	1	0.374429	0.1511
2	0.05573	0.280166	2	-0.54819	0.353775
3	-0.50956	0.201743	3	-0.13553	0.162221
4	0.012076	0.197373	4	-0.2018	0.158233
5	0.200264	0.362977	5	-0.39943	0.188178
6	-0.52701	0.250627	6	0.153642	0.363937
7	-0.1773	0.188311	7	0.156517	0.231125
8	-0.43907	0.184491	8	-0.33242	0.262342
9	0.0525	0.5	9	-0.18481	0.17975
10	-0.13846	0.5547	10	-0.79808	0.230817
11	-0.05767	0.350285	11	-1.59153	0.388955
12	-0.40615	0.392232			
13	-0.37973	0.217597			
14	-0.07692	0.158292			
15	0.165485	0.486217			

References (Appendix Table 1)

[1] C. S. Berkey, D. C. Hoaglin, F. Mosteller, and G. A. Colditz, “A random-effects regression model for meta-analysis,” *Stat. Med.*, vol. 14, no. 4, pp. 395–411, Feb. 1995.

[2] D. K. Bhaumik, A. Amatya, S.-L. Normand, J. Greenhouse, E. Kaizar, B. Neelon, and R. D. Gibbons, “Meta-Analysis of Rare Binary Adverse Event Data,” *J. Am. Stat. Assoc.*, vol. 107, no. 498, pp. 555–567, Jun. 2012.

[3] S. E. Brockwell and I. R. Gordon, “A comparison of statistical methods for meta-analysis,” *Stat. Med.*, vol. 20, no. 6, pp. 825–840, Mar. 2001.

[4] G. Knapp and J. Hartung, “Improved tests for a random effects meta-regression with a single covariate,” *Stat. Med.*, vol. 22, no. 17, pp. 2693–2710, Sep. 2003.

[5] E. Kontopantelis, D. A. Springate, and D. Reeves, “A Re-Analysis of the Cochrane Library Data: The Dangers of Unobserved Heterogeneity in Meta-Analyses,” *PLoS ONE*, vol. 8, no. 7, p. e69930, Jul. 2013.

[6] P. C. Lambert, A. J. Sutton, P. R. Burton, K. R. Abrams, and D. R. Jones, “How vague is vague? A simulation study of the impact of the use of vague prior distributions in MCMC using WinBUGS,” *Stat. Med.*, vol. 24, no. 15, pp. 2401–2428, Aug. 2005.

[7] P. W. Novianti, K. C. B. Roes, and I. van der Tweel, “Estimation of between-trial variance in sequential meta-analyses: A simulation study,” *Contemp. Clin. Trials*, vol. 37, no. 1, pp. 129–138, Jan. 2014.

[8] T. Panityakul, C. Bumrungsup, and G. Knapp, “On Estimating Residual Heterogeneity in Random-Effects Meta-Regression: A Comparative Study,” *J. Stat. Theory Appl.*, vol. 12, no. 3, p. 253, 2013.

[9] K. Sidik and J. N. Jonkman, “A comparison of heterogeneity variance estimators in combining results of studies,” *Stat. Med.*, vol. 26, no. 9, pp. 1964–1981, Apr. 2007.

[10] K. Sidik and J. N. Jonkman, “Simple heterogeneity variance estimation for meta-analysis,” *J. R. Stat. Soc. Ser. C Appl. Stat.*, vol. 54, no. 2, pp. 367–384, Apr. 2005.

[11] Y. Chung, S. Rabe-Hesketh, and I.-H. Choi, “Avoiding zero between-study variance estimates in random-effects meta-analysis,” *Stat. Med.*, vol. 32, no. 23, pp. 4071–4089, 2014.

[12] Y. Chung, S. Rabe-Hesketh, V. Dorie, A. Gelman, and J. Liu, “A Nondegenerate Penalized Likelihood Estimator for Variance Parameters in Multilevel Models,” *Psychometrika*, vol. 78, no. 4, pp. 685–709, Oct. 2013.

[13] W. Viechtbauer, “Bias and Efficiency of Meta-Analytic Variance Estimators in the Random-Effects Model,” *J. Educ. Behav. Stat.*, vol. 30, no. 3, pp. 261–293, Sep. 2005.

[14] B. J. Biggerstaff and R. L. Tweedie, “Incorporating variability in estimates of heterogeneity in the random effects model in meta-analysis,” *Stat. Med.*, vol. 16, no. 7, pp. 753–768, Apr. 1997.

[15] R. DerSimonian and N. Laird, “Meta-analysis in clinical trials,” *Control. Clin. Trials*, vol. 7, no. 3, pp. 177–188, Sep. 1986.

[16] R. DerSimonian and R. Kacker, “Random-effects model for meta-analysis of clinical trials: an update,” *Contemp. Clin. Trials*, vol. 28, no. 2, pp. 105–114, Feb. 2007.

[17] R. J. Hardy and S. G. Thompson, “A likelihood approach to meta-analysis with random effects,” *Stat. Med.*, vol. 15, no. 6, pp. 619–629, Mar. 1996.

[18] K. Sidik and J. N. Jonkman, “A note on variance estimation in random effects meta-regression,” *J. Biopharm. Stat.*, vol. 15, no. 5, pp. 823–838, 2005.

[19] K. Sidik and J. N. Jonkman, “Robust variance estimation for random effects meta-analysis,” *Comput. Stat. Data Anal.*, vol. 50, no. 12, pp. 3681–3701, Aug. 2006.

[20] S. G. Thompson and S. J. Sharp, “Explaining heterogeneity in meta-analysis: a comparison of methods,” *Stat. Med.*, vol. 18, no. 20, pp. 2693–2708, Oct. 1999.

[21] K. Thorlund, J. Wetterslev, T. Awad, L. Thabane, and C. Gluud, “Comparison of statistical inferences from the DerSimonian–Laird and alternative random-effects model meta-analyses – an empirical assessment of 920 Cochrane primary outcome meta-analyses,” *Res. Synth. Methods*, vol. 2, no. 4, pp. 238–253, Dec. 2011.

[22] W. Viechtbauer, “Confidence intervals for the amount of heterogeneity in meta-analysis,” *Stat. Med.*, vol. 26, no. 1, pp. 37–52, Jan. 2007.

[23] J. Sánchez-Meca and F. Marín-Martínez, “Confidence intervals for the overall effect size in random-effects meta-analysis,” *Psychol. Methods*, vol. 13, no. 1, pp. 31–48, Mar. 2008.

[24] U. Malzahn, D. Böhning, and H. Holling, “Nonparametric estimation of heterogeneity variance for the standardised difference used in meta-analysis,” *Biometrika*, vol. 87, no. 3, pp. 619–632, Sep. 2000.

[25] L. V. Hedges and I. Olkin, *Statistical methods for meta-analysis*. Orlando: Academic Press, 1985.

[26] J. Bowden, J. F. Tierney, A. J. Copas, and S. Burdett, “Quantifying, displaying and accounting for heterogeneity in the meta-analysis of RCTs using standard and generalised Q statistics,” *BMC Med. Res. Methodol.*, vol. 11, no. 1, p. 41, Apr. 2011.
